# Microenvironment Stiffness Amplifies Post-ischemia Heart Regeneration in Response to Exogenous Extracellular Matrix Proteins in Neonatal Mice

**DOI:** 10.3389/fcvm.2021.773978

**Published:** 2021-11-05

**Authors:** Xinming Wang, Valinteshley Pierre, Subhadip Senapati, Paul S.-H. Park, Samuel E. Senyo

**Affiliations:** ^1^Department of Biomedical Engineering, Case School of Engineering, Case Western Reserve University, Cleveland, OH, United States; ^2^Department of Ophthalmology and Visual Sciences, School of Medicine, Case Western Reserve University, Cleveland, OH, United States

**Keywords:** heart regeneration, extracellular matrix, decellularization, microenvironment stiffness, myocardial infarction, cell cycle activity

## Abstract

The cardiogenesis of the fetal heart is absent in juveniles and adults. Cross-transplantation of decellularized extracellular matrix (dECM) can stimulate regeneration in myocardial infarct (MI) models. We have previously shown that dECM and tissue stiffness have cooperative regulation of heart regeneration in transiently regenerative day 1 neonatal mice. To investigate underlying mechanisms of mechano-signaling and dECM, we pharmacologically altered heart stiffness and administered dECM hydrogels in non-regenerative mice after MI. The dECM combined with softening exhibits preserved cardiac function, LV geometry, increased cardiomyocyte mitosis and lowered fibrosis while stiffening further aggravated ischemic damage. Transcriptome analysis identified a protein in cardiomyocytes, CLCA2, confirmed to be upregulated after MI and downregulated by dECM in a mechanosensitive manner. Synthetic knock-down of CLCA2 expression induced mitosis in primary rat cardiomyocytes in the dish. Together, our results indicate that therapeutic efficacy of extracellular molecules for heart regeneration can be modulated by heart microenvironment stiffness *in vivo*.

## Introduction

Cardiovascular disease is the leading cause of death worldwide. World Health Organization (WHO) estimates 17.9 million people will die from cardiovascular diseases each year (1). Coronary heart disease (CHD) is the most common type of cardiovascular disease. Heart remodeling and irreversible scarring can permanently lower heart function in heart attack patients. In the worst scenario, the damage can develop to heart failure which currently can only be cured by heart transplantation. Because the therapies for myocardial infarction (MI) provide stabilization and partial recovery, the incidence of heart failure after an acute myocardial infarction is as high as 25% (2). Thus, promoting heart regeneration in post-ischemic hearts will benefit heart attack patients.

Adult mammalian hearts recover poorly from heart injury because of the extremely low proliferation of cardiomyocytes and permanent fibrosis. In contrast, neonatal rodents and pigs can fully recover from heart injury within 3 weeks (3–5). The robust heart regenerative capacity disappears within 3 days after birth. The mechanism underlying the heart regenerative capacity has not been fully realized, however, a natural decrease in cardiomyocyte proliferation with age and alternate immune responses are observed post-injury between regenerative and non-regenerative hearts (6, 7). Delivering biological molecules from regenerative hearts has been shown to benefit the heart post-injury response. Establishing molecular mechanisms could lead to new druggable targets for patients.

In addition to biological molecules that stimulate cardiomyocyte proliferation and reduce fibrosis, mechanical properties of heart have also been demonstrated to affect cardiac biology. Heart stiffness increases rapidly in new born animals coincident with loss of regeneration potential (8, 9). Post-MI fibrosis further increases stiffness and impairs cardiac structural organization (10, 11). Elastomeric substrates mimicking aging and disease-mediated stiffness lower primary cardiomyocyte proliferation (12–15). Mechanical unloading *in vivo* lowers fibrosis (8, 16). The mechanism of mechano-regulation in cardiomyocyte proliferation is not clear. Recent evidence of a pro-mitotic signaling axis, agrin–YAP pathway, links the ECM to nuclear signaling and sensitivity to microenvironment stiffness (17–19).

Decellularized extracellular matrix (dECM) derived biomaterial has been investigated for tissue engineering and regenerative medicine. The dECM contains insoluble fibrous molecules and soluble bioactive molecules (20–23). dECM hydrogel releases biomolecules locally that stimulate mitosis of neighboring cardiomyocytes. The dECM hydrogel derived from adult porcine hearts has been demonstrated to be biocompatible, safe, and promising for ischemic heart injury treatment in early clinical trials (24, 25). The dECM derived from regenerative zebrafish, neonatal mice, and fetal pig hearts improved heart post-injury response compared to allogenic adult heart dECM in mouse MI model (16, 26–28). The dECM treatment reduces fibrosis, increases vascularization, and stimulates cardiomyocyte cell cycle activity. The difference in dECM derived from early-age hearts compared to adult sourcing is possibly caused by the different components. Several extracellular proteins such as agrin, periostin, and neuregulin-1 have been reported to promote cardiomyocyte proliferation and angiogenesis (29–31). For example, the fraction of agrin and periostin in fetal heart dECM is significantly higher than adult dECM (21).

In this study, we investigated the effects of modulating the heart stiffness with dECM exogenous treatment on heart regeneration in a surgical MI model using low-regenerative juvenile mice. The dECM is derived from fetal (regenerative) and adult (non-regenerative) porcine hearts. We hypothesize that re-introduction of fetal extracellular biomolecules with tissue softening stimulates protection from post-MI damage toward heart regeneration. β-aminopropionitrile (BAPN), a lysyl oxidase inhibitor, was delivered systemically at low levels to lower collagen crosslinking and tissue stiffness. For comparative analysis, genipin, a plant-derived protein crosslinker, was delivered systemically to increase extracellular matrix crosslinking and stiffness. Permanent left coronary artery ligation was conducted on the mice followed by intra-myocardial injection of dECM in the hypoxic region. Cell cycle activity, fibroblast activation, fibrosis, and vascularization were examined on day 3 and day 23 post-MI. Heart function was examined by echocardiography at week 1 and 3 weeks post-MI. Using total mRNA sequencing data acquired after combined dECM and stiffness treatment, we identified a protein found to be upregulated in the ischemic heart, chloride channel accessory 2 (CLCA2). CLCA2 was observed to be sensitive to both microenvironment mechanical properties and dECM. Knocking down CLCA2 stimulated cell division of primary cardiomyocytes. Our results suggest that heart microenvironment stiffness modulates heart post-ischemic response induced by interaction with extracellular proteins.

## Materials and Methods

### dECM Preparation

Fetal or adult porcine ventricles from 3 to 4 animals were diced to ~125 mm^3^ cubes and washed in purified water to remove blood. Adult hearts were decellularized in 1% sodium dodecyl sulfate (SDS) (Millipore-Sigma, St. Louis, MO, US) for up to 4 days. Fetal hearts decellularized in 0.5% SDS. Cell removal was confirmed by discoloration and DAPI staining. Hearts were then washed in triton X-100 (Millipore-Sigma) solution for 4 h, 1% for adult heart and 0.5% for fetal heart. After washing in water 3 times over 12 h, decellularized hearts were lyophilized and stored at −80°C. To generate solubilized dECM, dECM was first pulverized by mortar and pestle in liquid nitrogen. The powder was weighed for 10 mg dry dECM to be digested by 1 mg pepsin (about 2,500 units/mg, Millipore-Sigma) at pH2. Adult dECM was digested for 36 h and fetal dECM was digested for 8 h both at room temperature on a magnetic stir plate. After digestion, solubilized dECM was neutralized by NaOH (Millipore-Sigma) and effective final concentration of 1× phosphate buffered saline (PBS) to reach a final pH of 7.4. Penicillin-streptomycin (P/S) (10,000 U/ml, Cytiva, Marlborough, MA, USA) was added to reach a final concentration of 100 U/ml. Solubilized dECM was stored at −20°C.

### Animals

All animals were handled according to Institutional Animal Care and Use Committee (IACUC) guidelines. Animal protocols were approved by Case Western Reserve University IACUC. All animals were maintained and housed under specific pathogen–free conditions at our animal facility accredited by the Association for Assessment and Accreditation of Laboratory Animal Care, International (AAALAC) at Case Western Reserve University. Both male and female mice were employed for this study.

### Left Coronary Artery Ligation and dECM Injection

Pregnant CD-1 IGS mice were purchased from Charles River Laboratories (USA) or bred internally to process litters. To generate stiffened hearts, genipin (Millipore-Sigma) was delivered by intraperitoneal injection at 10 mg/kg daily to neonatal mice from birth at P1 to P4. To generate soft hearts, BAPN (Millipore-Sigma) was delivered at 10 mg/kg daily. The postnatal myocardial infarction model followed established protocols with modifications (32–37). Juvenile (age P5) mice were anesthetized by hypothermia and then transferred to surgical mat resting on ice to maintain low temperature. After sanitizing skin with betadine and 70% ethanol, a lateral thoracotomy was made on the fourth intercostal space. Left coronary artery was visualized under stereo-microscope. Pericardium above left ventricle separated by blunt forceps. Left coronary artery was ligated by 10-0 nylon suture (AROSurgical, Newport Beach, CA, US). The ligation site was slightly above the root of left anterior descending artery. Immediately after ligation, 3 μl of solubilized adult or fetal dECM were injected into myocardium at two sites, one above and one below the ligation site, by 10 μl Hamilton syringe (Hamilton, Reno, NV, US) with a 32G needle. PBS buffer was injected in MI-control animals. No ligation was applied to sham animals after pericardium dissection. The intercostal incision was closed by 6-0 polypropylene suture (Surgical Specialties, Wyomissing, PA, US). Skin was closed by skin-glue (Henry Schein, Melville, NY, US). Neonates were warmed up and returned to littermates after recovering from anesthesia. After surgical procedures were completed, the littermates were returned to dame.

### Echocardiography and BrdU Labeling

Heart function was measured by a Vevo 3100 preclinical imaging system (Fujifilm VisualSonics, USA) equipped with a MX550D transducer. Week 1 and 3 post-MI mice were anesthetized by 4% isoflurane (Patterson veterinary, Greeley, CO, USA) and maintained by 1% isoflurane during the recording. M-mode and B-mode were recorded at long- and short-axis planes, respectively. Heart rate was maintained at 400–500 beats per minute. Ejection fraction, fractional shortening, cardiac output, and stroke volume were measured by Vevo lab 2.1.0 (VisualSonics).

Bromodeoxy uridine (BrdU; Millipore-Sigma) was dissolved in 1× PBS and sterilized with a 0.22 μm syringe filter. BrdU was injected to mice at 0.1 mg/g body weight. For mice euthanized on day 3 post-MI, one dose of BrdU was delivered 12 h before sacrifice. For mice euthanized at week 3 post-MI, BrdU was injected daily for 2 days before sacrifice.

### Atomic Force Microscopy

Atomic force microscopy (AFM) was used to measure the elastic modulus of the acellular tissue matrix after decellularization of mouse ventricle hearts from animal experiments after BAPN and genipin treatment. Juvenile mouse ventricles were decellularized and adhered to 35 mm Petri dishes using a thin layer of fast-drying adhesive. Elastic modulus measurements were conducted using a Keysight 5500 AFM (Keysight Technologies, Santa Rosa, CA) equipped with an infrared laser, using PicoView 1.20 software. Silicon nitride tips (DNP-S, Bruker Corporation, Camarillo, CA) with a nominal spring constant of 0.06 N/m were used for AFM experiments. Samples were immersed in 1× PBS solution during the AFM measurements and force-distance (FD) curves were collected at random positions on the immobilized samples by applying a force of 1 nN with a tip speed of 6.25 μm/s. Three different sets of samples were analyzed for control and experimental (BAPN and genipin) mice. A Hertzian model modified for pyramidal tips was used for fitting approach force-distance curves to determine the Young's modulus, using a plug-in package from Keysight Technologies (pico-café.com), as described previously (38).

### Immunohistochemistry

Heart tissue was fixed in 4% paraformaldehyde solution for 3 days at 4°C. Then tissues were washed in 1× PBS and balanced in 10 and 20% sucrose solution for 1 h and in 30% sucrose solution overnight at room temperature on a rotator. Samples were then embedded in OCT (Tissue-Tek, Torrance, CA, US) in plastic molds and froze overnight at −80°C. Samples were sectioned to 5 μm slides and stored at −80°C before use.

Slides were thawed at room temperature and washed in 1 x tris-buffered saline (TBS) for 3 x 5 min. Then samples were circled by a hydrophobic pen. Samples were incubated in 20–30 μl 0.1% triton x-100 in TBS (washing buffer) for 3 x 5 min, and then incubated in 30 μl 10% goat serum in washing buffer (blocking buffer) for 1 h at room temperature. After removing blocking buffer, samples were incubated in 5 μg/ml primary antibody staining buffer (1% bovine serum albumin in washing buffer) overnight at 4°C. Chicken anti-vimentin (Abcam, Waltham, MA, US), rabbit anti-platelet derived growth factor receptor-α (Pdgfr-α) (Abclonal, Woburn, MA, US), rabbit anti-α-SMA (Cell Signaling, Danvers, MA, US and ThermoFisher, Waltham, MA, US), mouse anti-troponin T (Developmental Studies Hybridoma Bank, Iowa City, IA, US), rabbit anti-BrdU (ThermoFisher), rabbit anti-Ki67 (ThermoFisher), rabbit anti-phospho-histone H3 (Abcam), and rabbit anti-CD31 (Abcam) antibodies were used. The final primary antibody concentration is 3–5 μg/ml. Samples were then washed in washing buffer 3 x 5 min and incubated in secondary antibody staining buffer (1% bovine serum albumin in washing buffer) for 1 h at room temperature. Fluorophore-conjugated goat-derived secondary antibodies (ThermoFisher) were used at a final concentration of 10 μg/ml. Samples were then stained by 0.3 μg/ml DAPI solution for 10 min and washed in 1 x TBS 3 x 5 min. Samples were immersed in aqueous mounting media (Vector Lab, Burlingame, CA, US) and sealed by nail polish (OPI, Calabasas, CA, US).

Masson's Trichrome staining was applied to visualize fibrotic regions. Slides were thawed at room temperature and washed in distilled water 3 x 5 min. Staining was then performed following the manufacturer protocol (Electron Microscopy Sciences, Hatfield, PA, US).

Fluorescent immunostained heart sections were imaged using a Zeiss Observer. Z1 microscope (Zeiss, White Plains, NY, US). MI groups' cardiomyocytes in the border area were imaged (within 250 μm from the edge of infarct area). Sham group's cardiomyocytes were randomly imaged in left ventricle wall. Fibroblasts in infarct and border areas were imaged (within 100 μm of collagen-rich infarct area and in the infarct area). Sham groups fibroblasts were randomly imaged in left ventricle wall. MI groups' blood vessels in the infarct and border areas were imaged (within 400 μm from the edge of collagen-rich infarct area). Sham group blood vessels were imaged randomly in the left ventricle wall. For each section, 5 images were randomly taken in the area mentioned above. Cell numbers were counted in imageJ by thresholding using *Auto Threshold* (Moments, Otsu, or Percentile methods). The segmented color channel of DAPI staining and the color channel of specific cell markers were overlayed to highlight specific cells of interest. Cell number was then counted using *Analyze Particle* function. BrdU, Ki67, Phh3, and α-SMA positive cells were counted manually.

Masson's Trichrome staining sections were imaged using an Olympus IX81 microscope (Olympus, Center Valley, PA, US) with automated stage. Four images were stitched to one picture to show the whole heart. The whole heart area was measured using imageJ by Auto thresholding. Fibrotic area was measured by Auto thresholding and manually choosing the infarct region.

### Western Blot

Mouse ventricles in cell lysis buffer (ThermoFisher) supplemented with protease inhibitor (Roche, USA), phosphatase inhibitor (Roche), and 1 mM phenylmethylsulfonyl fluoride (ThermoFisher) were homogenized by bead blender (Next Advance, Troy, NY, US). Tissue lysate was collected after centrifugation. Protein concentration measured by BCA assay (ThermoFisher). Measured lysate (20 μg) was loaded to each lane of a 15-well 4–20% polyacrylamide gel (Bio-Rad, Hercules, CA, US). After electrophoresis, proteins were transferred to 0.45 μm nitrocellulose membrane (Bio-Rad) using semi-dry transferring. Membranes were rinsed in 0.15% tween-20 with TBS (WB washing buffer) and blocked in 4% non-fat dry milk in WB washing buffer at 4°C overnight. Membranes were then washed 3 x 5 min in WB washing buffer and incubated with the primary antibody in staining buffer (4% BSA in washing buffer) at room temperature for 2 h. Rabbit anti-CLCA2 (ProteinTech, Rosemont, IL, US), mouse anti-GAPDH (ProteinTech), mouse anti-HSC70 (Santa Cruz Biotechnology, Dallas, TX, US) were used following the dilutions recommended by manufacturers. Membranes were then washed in WB washing buffer 3 x 5 min, and incubated in goat-derived horseradish peroxidase-conjugated secondary antibodies (Cell Signaling) for 1 h at room temperature. Membranes were then washed in WB washing buffer and imaged immediately. SuperSignal West Femto (ThermoFish) was used for chemiluminescent imaging. Bands intensities were measured by ImageJ.

### Cardiomyocyte Isolation and RNA Interference

Primary rat ventricle cardiomyocytes were isolated from Sprague Dawley day 1 neonatal rats (Charles River) using the Neonatal Heart Dissociation Kit, mouse, and rat (Miltenyi Biotec, USA). Percoll gradient centrifuge was used to separate cardiomyocytes and non-cardiomyocytes. Cardiomyocytes were plated to collagen type I coated 8 mm coverslips in 48-well tissue culture plate at 21,000 cells per cm^2^. Cells were cultured in 10% FBS and 100 U/ml P/S in DMEM media overnight. After PBS wash, 5% FBS DMEM (without antibiotic) was added to each well. Following the manufacturer protocol, CLCA2 siRNA (ThermoFisher) and lipofectamine 3000 (ThermoFisher) were mixed in M199 media. The siRNA-lipofectamine mixture was added to each well to reach a final CLCA2 siRNA concentration of 200 nM. Cells were transfected for 48 h. After transfection, the coverslips for fetal dECM treatment were transferred to wells containing pre-cast fetal dECM hydrogel. Cells were cultured in no-serum DMEM for 24 h. Cells were then cultured in no-serum DMEM containing 10 μM BrdU for 6 h. Cells were fixed in 4% PFA and processed for immunostaining.

To assess protein expression after 48 h siRNA transfection, cells plated in 12-well plates were incubated in lysis buffer for subsequent western blot analysis as described above.

### Statistical Analysis

All data were analyzed using Prism 7 (GraphPad). Statistical analysis between 2 groups used two-tailed *t*-test of 95% confidence level and Gaussian distribution of experimental points. Analysis between 3 or more groups used one-way ANOVA with Gaussian distribution. Multiple comparisons in one-way ANOVA analysis were conducted using Tukey test with 95% confidence interval. Two-way ANOVA was used for comparing multiple independent experimental stimuli (i.e., altered stiffness and dECM treatments). All data are presented as mean ± standard deviation. For cardiomyocyte, in 3 days post-MI animals, 400–600 cardiomyocytes per animal, or 2,000–3,000 cardiomyocytes per treatment were analyzed; in 3 weeks post-MI animals, 200–400 cardiomyocytes per animal, or 1,000–2,000 cardiomyocytes per treatment were analyzed. For fibroblasts, in 3 days post-MI animals, 30–100 fibroblasts per animal, or 150–500 fibroblasts per treatment were analyzed; in 3 weeks post-MI animals, 50–200 fibroblasts, or 250–1,000 fibroblasts per treatment were analyzed.

## Results

### Lowering Heart Stiffness Enhances the Protective Effects of Fetal dECM on Heart Function

**Figure 1:**
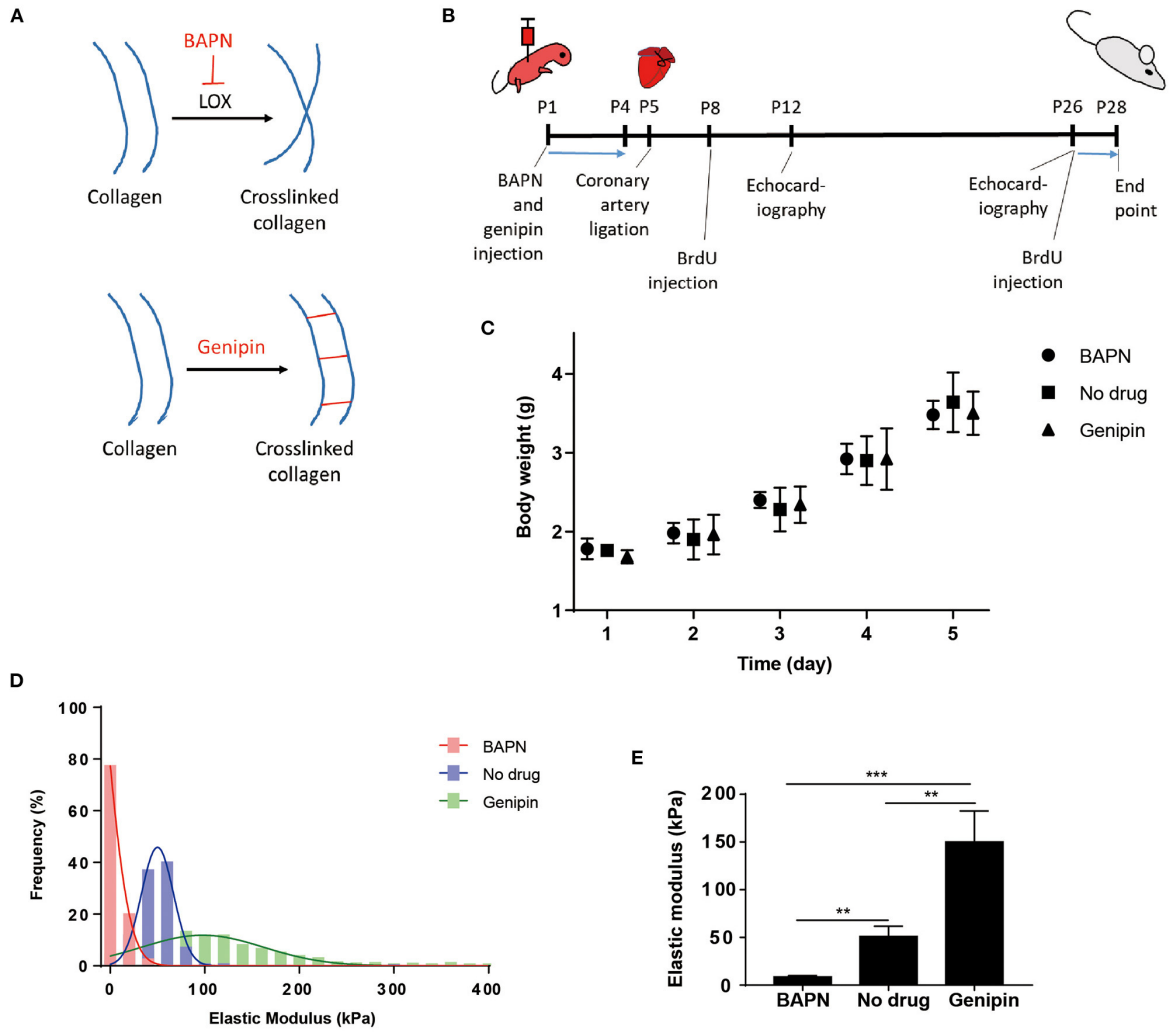
Systemic delivery of BAPN and genipin changes neonatal mouse heart stiffness. **(A)** BAPN decreases microenvironment stiffness by inhibiting lysyl oxidase crosslinking of collagen. Genipin increases stiffness by crosslinking fibrous proteins. **(B)** Schematic of the experimental protocol. **(C)** Body weights of animals during the 4 days BAPN and genipin treatments. **(D)** Distribution of all AFM measurements in each treatment group. **(E)** BAPN treatment of juvenile mice decreased the average stiffness of the decellularized heart from 50.9 to 8.6 kPa. Genipin increased the average decellularized heart stiffness to 150.1 kPa. [**(D,E)** Three hearts per group; 200–300 spots per heart, one-way ANOVA and Tukey's test, **p < 0.01, ***p < 0.001. **(C)** Five to six hearts per group. Data represented as mean ± standard deviation].

The elastic modulus of the cardiac extracellular matrix was measured by atomic force microscopy (AFM) after *in vivo* treatments with BAPN and genipin. BAPN irreversibly inhibits the activity of lysyl oxidase (LOX) which crosslinks collagen fibers (Figure 1A). Genipin, on the other hand, crosslinks proteins in extracellular matrix. BAPN and genipin were injected to mice (Figure 1B). BAPN and genipin treatments at low effective concentrations for 4 days did not significantly change body weights of mice (Figure 1C). In addition, echocardiography of sham hearts in P12 (Supplementary Figure 1A) and P26 (Supplementary Figure 1B) mice indicates that BAPN and genipin treatments did not change left ventricle end systolic and diastolic diameters. The similar P28 body weights across groups suggests that the treatments did not significantly affect juvenile mice development (Supplementary Figure 1C). AFM measurements (700–900 points each group) of decellularized ventricles after the two individual treatments indicated that BAPN decreased the average stiffness of the heart extracellular matrix from 50.9 ± 10.8 to 8.6 ± 1.3 kPa, and genipin increased the average stiffness to 150.1 ± 32.4 kPa (Figures 1D,E).

**Figure 2:**
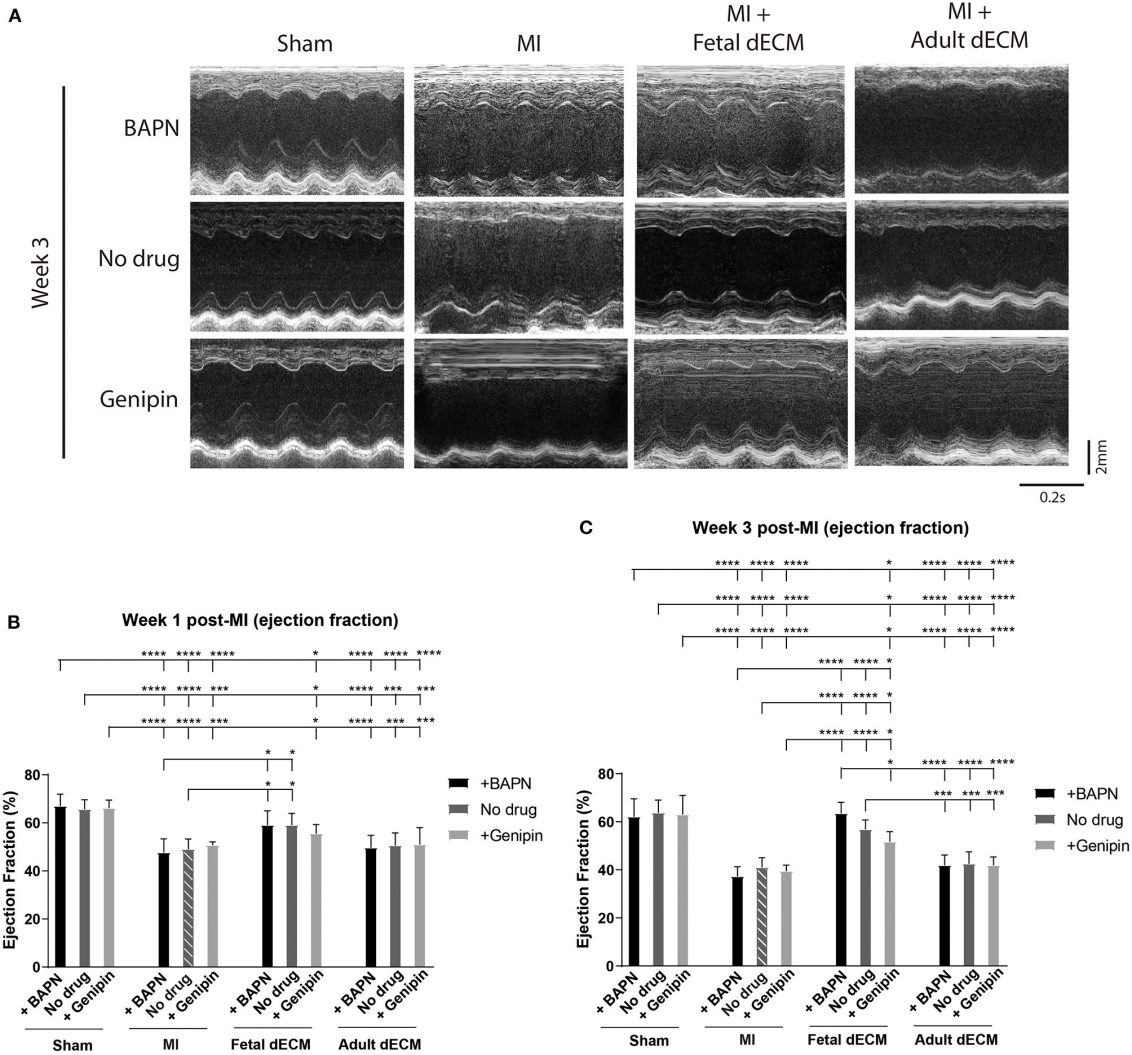
Increasing heart stiffness reduced cardiac function in fetal dECM treated hearts. **(A)** Cardiac function measured by echocardiography. **(B)** Ejection fraction at week 1 post-MI. Fetal dECM treated mice showed a higher ejection fraction at week 1 post-MI compared to MI-no drug. **(C)** Ejection fraction at week 3 post-surgery. Fetal dECM protection of ejection fraction was more pronounced at week 3 with BAPN-fetal dECM treated (softened) hearts showing the highest ejection fraction. Adult dECM treatments did not alter ejection fraction even when combined with tuning stiffness. [**(B,C)** *n* = 5, two-way ANOVA and Tukey's test, *p < 0.05, ***p < 0.001, ****p < 0.0001. Data represented as mean ± standard deviation. The lines above the plot represent the statistical significance between the tick-designated first group with the following ticks].

Permanent coronary artery ligation results in extensive fibrosis and loss of cardiac function in adult mice models and to a comparable degree in juvenile mice, such as P5 mice employed in this study (4, 39). The heart injury was consistent as indicated by left ventricle end diastolic and systolic diameters in MI hearts measured by echocardiography at week 1 post-surgery (Supplementary Figure 1D) and by histochemistry for scar tissue on day 3 post-surgery (Supplementary Figure 3). Both male and female mice were included in the experiments. Cardiac function was measured by echocardiography at week 1 and 3 post-MI and the MI group showed a characteristic loss of function approaching 20% compared to sham juvenile mice (Figure 2A). Tuning heart stiffness did not alter cardiac function in sham and MI mice. MI hearts treated with fetal dECM and fetal dECM combined with BAPN (decreased heart stiffness) showed protection early at week 1 that became significantly more pronounced at week 3 compared with the MI groups including improved ejection fraction (Figures 2B,C), fractional shortening (Supplementary Figures 2A,B), stroke volume (Supplementary Figure 2C), and cardiac output (Supplementary Figure 2D). In fetal dECM treated hearts, genipin-stiffened subgroups showed decreased cardiac functions compared to BAPN-softened subgroups at week 3. Adult dECM treatment for MI did not alter ejection fraction nor fractional shortening compared to the MI group. However, adult dECM treatments on a stiffened heart (150.1 kPa, genipin-treated hearts) showed a lower stroke volume than on a softened heart (8.6 kPa, BAPN-treated hearts) (Supplementary Figure 2C). Together, the results show that cardiac stiffness affects the efficacy of exogenous extracellular matrix therapy on ischemic heart injury.

### Softening Heart and Fetal dECM Reduced Fibrosis and Fibroblast Activation

**Figure 3:**
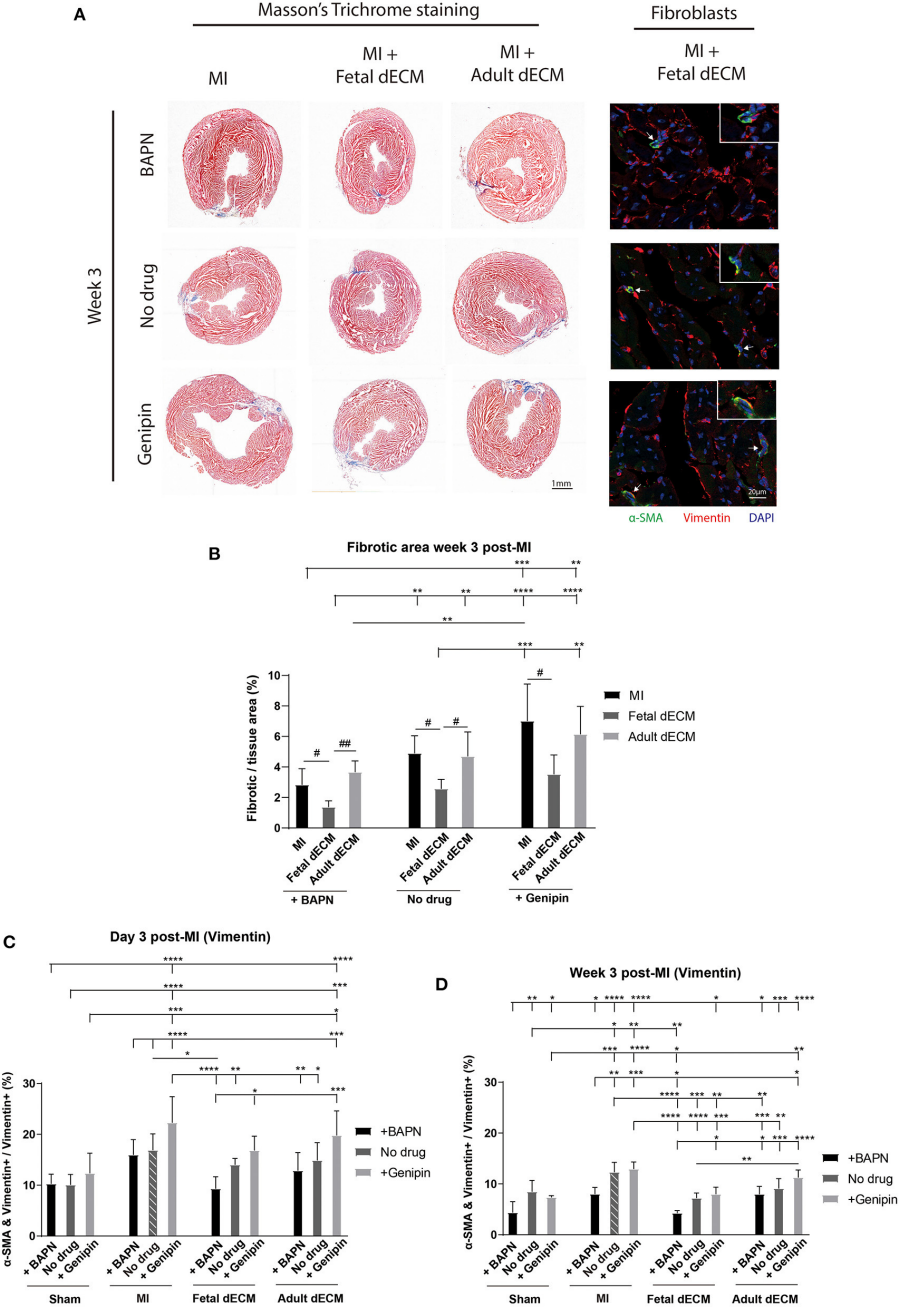
Decreasing heart stiffness and dECM treatments lowered cardiac fibrosis and fibroblast activation at week 3 post-surgery. **(A)** Masson's trichrome staining was used to examine the fibrotic tissue in hearts. Fibroblast activation was examined by immunostaining for vimentin and α-SMA. **(B)** Fibrotic area in MI hearts with tuning of stiffness at week 3 post-surgery. Stiffening tissue indicated a trend of increasing fibrosis in MI groups. Fetal dECM treatment reduced fibrosis compared to MI-control and adult dECM treatment in BAPN-treated and no drug treated hearts, but not in genipin-treated hearts. Adult dECM did not significantly change fibrosis compared to MI-control. **(C)** Day 3 quantification of α-SMA and vimentin double positive cells in dECM treatment groups. Combined treatment of fetal dECM and BAPN lowered fibroblast activation compared to MI-no drug on day 3. Softening by BAPN showed a trend of lowering fibroblast activation in dECM treated hearts. **(D)** Week 3 quantification in dECM treated hearts. Fetal dECM reduced fibroblast activation compared to MI-no drug in all stiffness conditions at week 3 post-surgery. Decreasing stiffness by BAPN lowered fibroblast activation in fetal dECM treated hearts compared to genipin-stiffening. [**(B)** *n* = 5, one-way ANOVA and Tukey's test for intragroup comparison (same stiffness), #*p* < 0.05, ##*p* < 0.01; two-way ANOVA and Tukey's test for intergroup comparisons, **p < 0.01. ***p < 0.001, ****p < 0.0001. **(C,D)** *n* = 5, two-way ANOVA and Tukey's test, *p < 0.05, **p < 0.01, ***p < 0.001, ****p < 0.0001. Data represented as mean ± standard deviation. The lines above the plot represent the statistical significance of the tick-designated initial group with the following ticks. The line without ticks represents the statistical significance of only two groups].

Juvenile mice exhibit permanent scarring that impairs function though without the dilation observed in adult MI models. Thus, large scar tissue and wall thinning were not expected. Heart fibrosis was examined by Masson's Trichrome staining. Fibrotic tissue can be visualized at week 3 post-MI (Figure 3A) while absent on day 3 post-MI (Supplementary Figure 3A). Changing tissue stiffness (BAPN and genipin) did not significantly change the fibrotic area (Figure 3B). However, a trend of lowered fibrosis was observed in softening heart without dECM treatments (MI). Fetal dECM treatment significantly lowered fibrotic area compared to MI hearts at all stiffness levels. Fetal dECM treatment also decreased the area of fibrotic tissue compared to adult dECM treatment in BAPN-softened and no drug treated hearts. Adult dECM treatments did not affect fibrosis directly. The results indicate that the fetal dECM with BAPN-mediated tissue softening show independent effects on lowering cardiac fibrosis.

Fibroblasts were evaluated in MI hearts to determine if tissue stiffness and dECM modulated fibrosis by lowering fibroblast activation. Fibroblast activation was examined by α-SMA expression in vimentin positive cells (Figure 3A). As expected, fetal dEM with BAPN softening lowered fibroblast activation compared to MI-no drug on day 3 (Figure 3C) and at week 3 (Figure 3D) post-surgery. Increasing heart stiffness with genipin lowered fetal dECM effects. Adult dECM did not significantly decreased fibroblast activation compared to MI-no drug on day 3, but lowered fibroblast activation at week 3 with BAPN softening. Fibroblast activation was also examined by co-staining for α-SMA and Pdgfr-α (Supplementary Figure 4A). Using Pdgfr-α avoids labeling infiltrating neutrophils and lymphocytes likely overlap with the larger initial vimentin positive population. BAPN softening decreased fibroblast activation in MI groups post-surgery. Fetal dECM significantly lowered fibroblast activation on day 3 (Supplementary Figure 4B) and at week 3 (Supplementary Figure 4C) post-surgery compared to MI-no drug. BAPN softening indicated a trend of increasing the fetal dECM effects. Adult dECM did not significantly change fibroblast activation compared to MI-no drug except with BAPN-softening in week 3 post-surgery hearts. These results suggest that fetal dECM and heart stiffness play complementary roles in regulating fibrosis through reduced fibroblast activation.

### Softening Heart Increases Fetal dECM Induced Cardiomyocyte Cell Cycle Activity

**Figure 4:**
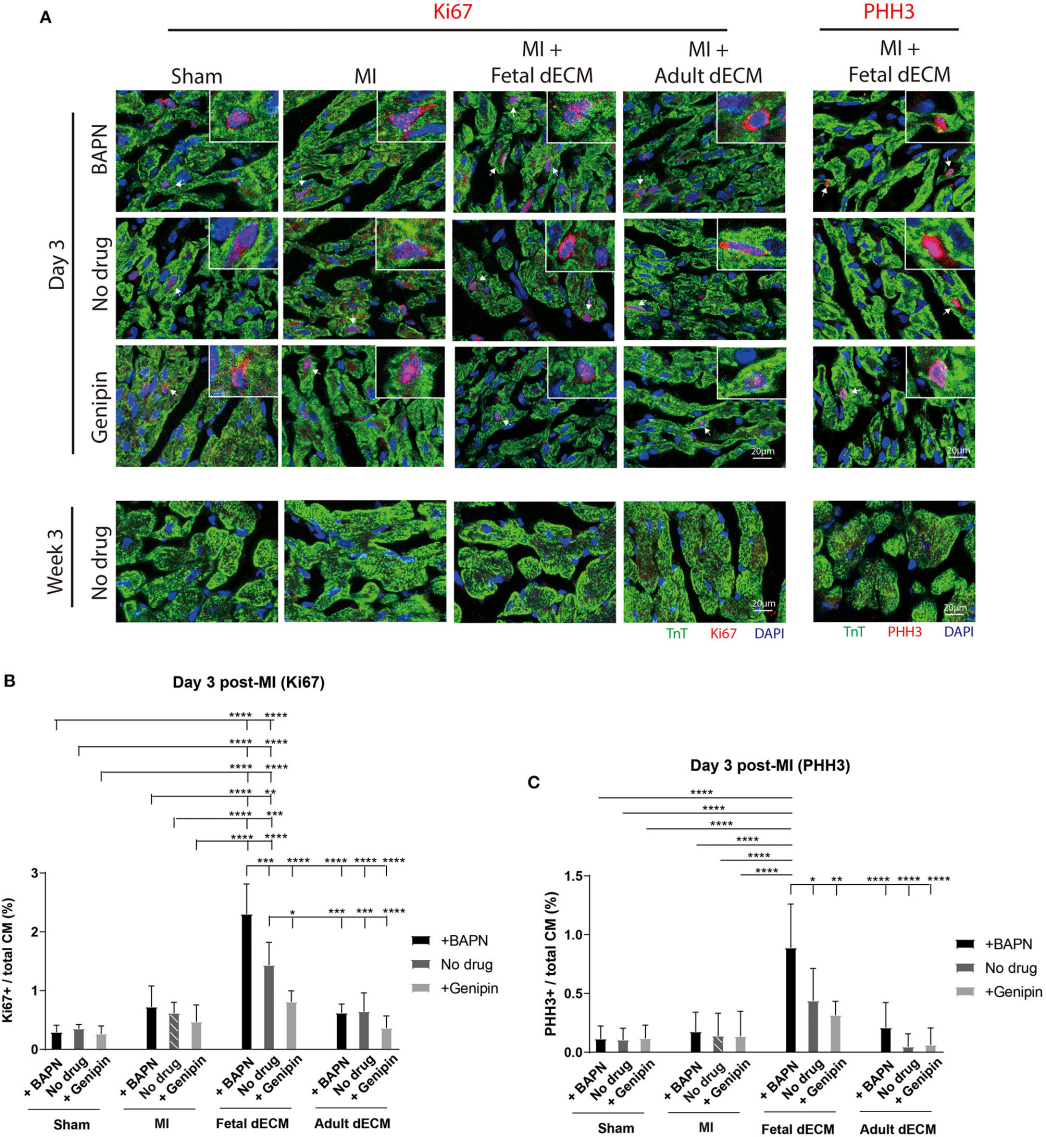
Fetal dECM treatment increases cardiomyocyte cell cycle activity in the MI heart. **(A)** Cardiomyocyte cell cycle activity on day 3 and at week 3 post-surgery was examined by immunostaining for Ki67, PHH3, and TnT. **(B)** Ki67 positive cardiomyocytes on day 3 post-surgery. Changing heart stiffness did not modulate Ki67 positive cardiomyocyte number in sham and MI. Fetal dECM and BAPN softening independently and synergistically increased Ki67 expression in cardiomyocytes compared to MI-control on day 3 post-surgery. Adult dECM treatment did not significantly change cardiomyocyte Ki67 expression. **(C)** PHH3 positive cardiomyocyte number on day 3 post-surgery. Fetal dECM increased PHH3 positive cells population in BAPN-softened hearts compared to MI-control. Increasing stiffness lowered PHH3 positive cells population in fetal dECM treated animals. [**(B,C)** *n* = 5, two-way ANOVA and Tukey's test, *p < 0.05, **p < 0.01, ***p < 0.001, ****p < 0.0001. Data represented as mean ± standard deviation. The lines above the plot represent the statistical significance of the tick-designated initial group with the following ticks. The line without ticks represents the statistical significance of only two groups].

Cardiomyocyte cell cycle activity was evaluated to determine if stiffness and dECM affect cardiomyocyte proliferation using cell cycle markers Ki67 (Figure 4A), PHH3 (Figure 4A), and BrdU labeling (Supplementary Figure 5A). Changing heart stiffness did not affect Ki67 expression in sham, MI, and adult dECM hearts on day 3 and at week 3 post-surgery. Fetal dECM treatment in BAPN-softened and MI hearts significantly increased Ki67 positive cardiomyocytes on day 3 post-surgery compared to MI-no drug hearts (Figure 4B) but not at week 3 (Supplementary Figure 5D). Decreasing heart stiffness (BAPN-treatment) promoted Ki67 positive cardiomyocytes in fetal dECM treated hearts. Similar effects were also observed in PHH3. Fetal dECM with BAPN-treatments significantly increased PHH3 positive cardiomyocytes compared to MI-no drug on day 3 post-surgery (Figure 4C). Unlike Ki67 (Supplementary Figure 5D) and PHH3 (Supplementary Figure 5E) at week 3 post-surgery, BrdU incorporation was increased in fetal dECM treatment with BAPN softening compared to MI-no drug on day 3 (Supplementary Figure 5B) and at week 3 (Supplementary Figure 5C) post-surgery likely reflecting the longer duration of labeling mitotic events. The results demonstrate that fetal dECM stimulates cardiomyocyte cell cycle activity at early post-injury stage. In addition, mechanical properties of the microenvironment can modulate fetal dECM induced cardiomyocyte cell cycle activity.

**Figure 5:**
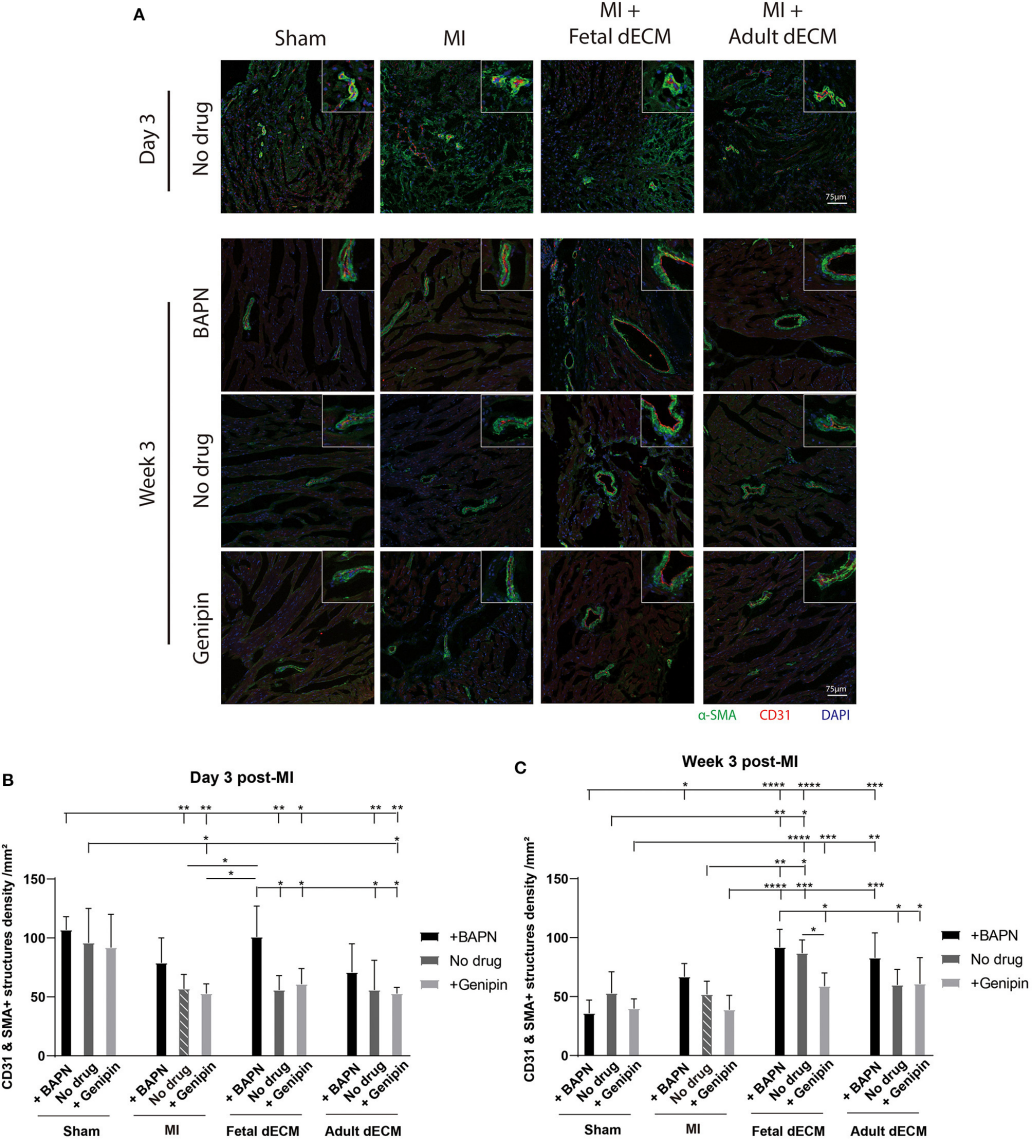
Lowering heart stiffness increases angiogenesis induced by dECM. **(A)** Vessel density was examined by immunostaining for CD31 and α-SMA. The number of CD31 and α-SMA double positive structures was counted. **(B)** Vessel density on day 3 post-surgery. Fetal dECM treatment increased vessel density in BAPN-softened hearts. Only BAPN and fetal dECM co-treated hearts showed higher angiogenesis compared to MI-no drug. A trend of increasing vessel density was observed in softening MI hearts. **(C)** Week 3 quantification of angiogenesis. Fetal dECM treatment increased angiogenesis in comparison to MI-no drug. Increasing heart stiffness by genipin reduced the effectiveness of fetal dECM treatments for angiogenesis. [**(B,C)** *n* = 5, two-way ANOVA and Tukey's test, *p < 0.05, **p < 0.01, ***p < 0.001, ****p < 0.0001. Data represented as mean ± standard deviation. The lines above the plot represent the statistical significance of the tick-designated initial group with the following ticks. The line without ticks represents the statistical significance of only two groups].

Angiogenesis was examined for evidence that new blood vessels were formed to support newly generated cardiomyocytes. Blood vessel density was evaluated by immunostaining for α-SMA and CD31 double positive structures (Figure 5A). Tuning heart stiffness did not affect the density of CD31 and α-SMA positive structures in sham hearts. A trend of increasing blood vessel density was observed in softening hearts in MI group. A significantly increased blood vessel density was observed in fetal dECM treatments compared to MI-no drug at week 3 post-surgery. Lowering heart stiffness in combination with fetal dECM treatment increased vessel density on day 3 (Figure 5B) and at week 3 (Figure 5C) in comparison to MI-no drug. The results indicate that fetal dECM and softening independently potentiate angiogenesis in the MI heart and combined play a complementary role in increasing angiogenesis density.

### Fetal dECM Lowers CLCA2 Protein Levels

In order to identify the signaling pathways that regulate the mechanosensitivity of fetal dECM treatment for heart regeneration, a mouse ventricle explant model was used to analyze transcriptome changes due to combined treatments of dECM and mechano-modulation for differentially expressed genes, as previously described (Supplementary Figure 6A) (16). The previous analysis was focused on fibroblast signaling; here, candidate regulators of cardiomyocyte proliferation were investigated. Reactome analysis indicated that a majority of fetal dECM down-regulated genes (compared to control) represented extracellular matrix fiber proteins and cell-matrix binding (Supplementary Figure 6B). The fetal dECM up-regulated genes are related to various signaling pathways including immune response, molecule transportation, and Rho family GTPases (Supplementary Figure 6C). The results suggest that cell-microenvironment interactions in cardiac tissue are affected by fetal dECM treatment.

The protein coding-genes that were inversely expressed in softened- or stiffened-fetal dECM treated explants were chosen for further analysis. The protein localization and function of the genes were determined by literature review. Low-abundant genes (FPKM <1) were excluded. The protein expression of 8 genes in hearts were examined by western blot. A protein, chloride channel accessory 2 (CLCA2) was screened from the differentially expressed genes (CLCA2: low-stiffness fetal vs. untreated *p* < 0.0005, medium-stiffness fetal vs. untreated *p* > 0.05.) from the transcriptome dataset related to cytoskeleton and cell-matrix binding with potential expression in cardiomyocytes. CLCA2 is a chloride ion channel regulator involved in cancer cell adhesion, migration, and proliferation (40–44). CLCA2 protein has not been investigated in cardiomyocytes though it regulates cell proliferation through multiple signaling pathways across different cell types (44). Thus, CLCA2 was interrogated for further analysis for a novel role in cardiac biology.

**Figure 6:**
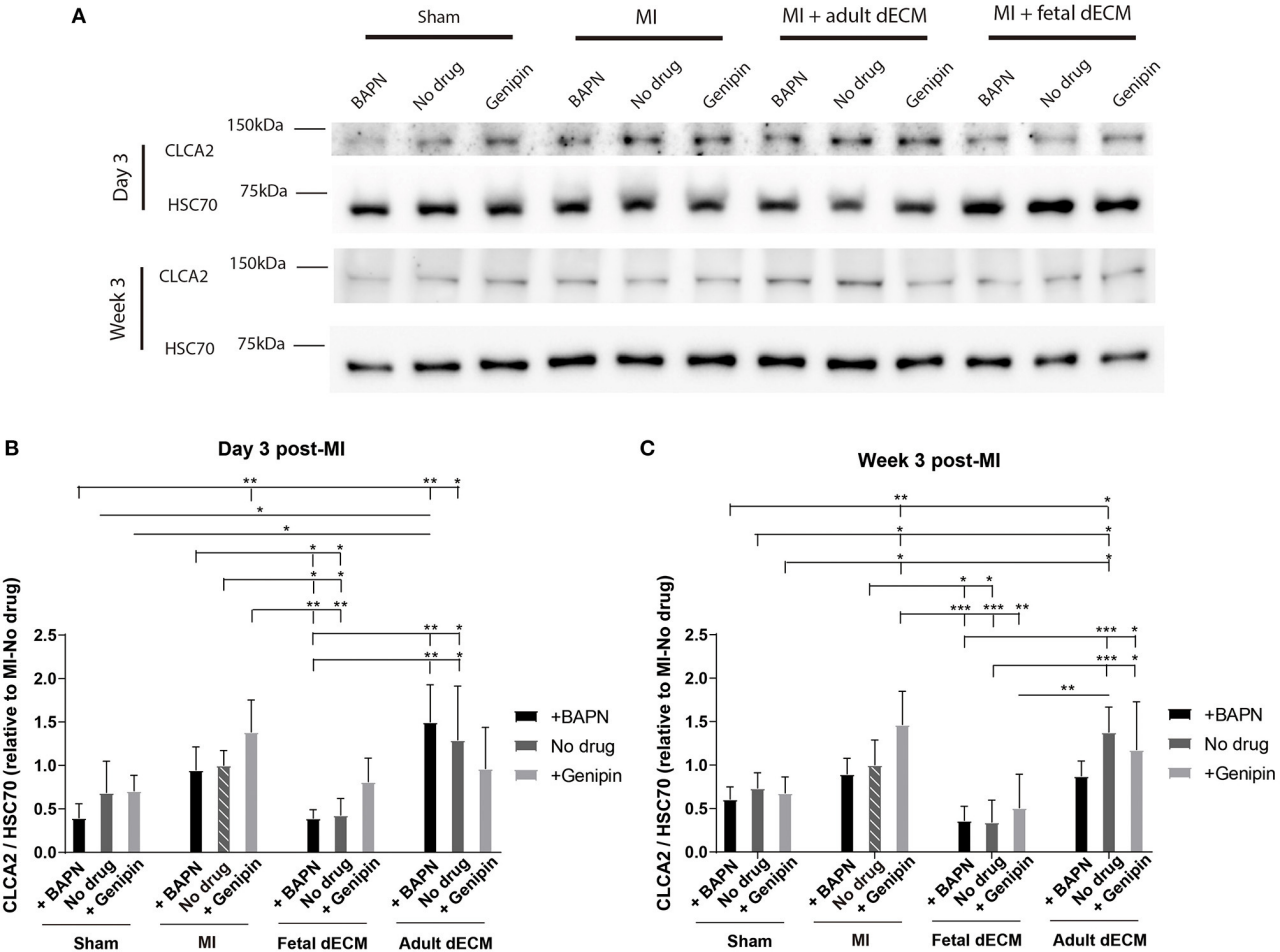
CLCA2 protein expressions were modulated by tissue stiffness and fetal dECM. **(A)** CLCA2 expression on day 3 and at week 3 post-surgery was examined by western blot. **(B)** CLCA2 levels on day 3 post-surgery. Changing heart stiffness did not significantly modulate CLCA2 levels in sham and MI hearts. Fetal dECM lowered CLCA2 expression in all conditions except with stiffening. **(C)** CLCA2 levels at week 3 post-surgery. Fetal dECM lowered CLCA2 expression in softened and untreated hearts compared to MI-no drug. [**(B,C)** *n* = 4, two-way ANOVA and Tukey's test, *p < 0.05, **p < 0.01, and ***p < 0.001. Data represented as mean ± standard deviation. The lines above the plot represent the statistical significance of the tick-designated initial group with the following ticks. The line without ticks represents the statistical significance of only two groups].

The distribution of CLCA2 in cardiac cells was examined by immunostaining (Supplementary Figure 7). CLCA2 primarily localizes in cardiomyocytes. CLCA2 protein expression was then examined by western blot to evaluate impact of dECM and stiffness (Figure 6A). CLCA2 expression increases with MI and shows mechanosensitivity post-ischemia (and not in sham groups) with a higher CLCA2 level observed in genipin-stiffened MI hearts but not in no drug and BAPN-softened MI hearts compared to sham hearts at week 3 (Figure 6C). Adult dECM treatment showed a noticeable trend for upregulating CLCA2 expression post-MI by day 3 (Figure 6B). Fetal dECM treatment significantly protected against MI-induced upregulation of CLCA2 expression evaluated on day 3 and at week 3 post-surgery. Stiffening (+genipin) the fetal dECM treated hearts reduced the protective effects with CLCA2 levels returning to the levels in the MI-no drug hearts. Together, the results suggest that fetal dECM reduces CLCA2 expression and the effect can be attenuated by stiffening heart in post-ischemic cardiac tissue.

### Interfering CLCA2 Expression Increases Cardiomyocyte Cell Cycle Activity *in vitro*

**Figure 7:**
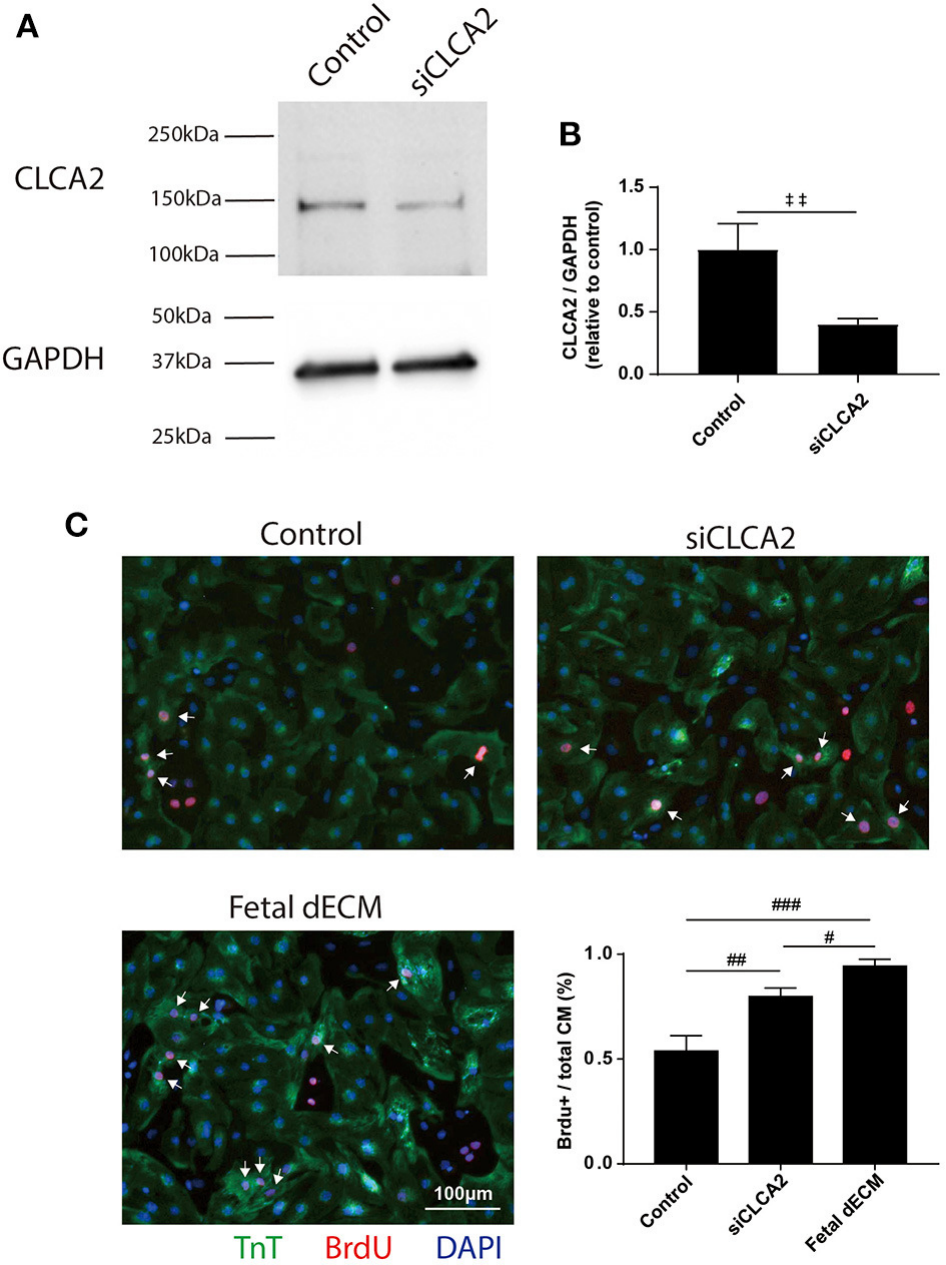
Decreasing CLCA2 expression promotes cardiomyocyte cell cycle activity. **(A)** CLCA2 protein levels in cardiomyocytes isolated from day 1 rats were decreased by CLCA2 siRNA. **(B)** CLCA2 protein level was reduced by about 60% after 48 h CLCA2 siRNA transfection. **(C)** Cardiomyocyte cell cycle activity was examined after CLCA2 knock-down by probing for BrdU incorporation. CLCA2 knock-down increased cardiomyocyte cell cycle activity compared to control. [**(B)** *n* = 3, *t*-test, ‡‡*p* < 0.01. **(C)** *n* = 3, one-way ANOVA and Tukey's test. #*p* < 0.05, ##*p* < 0.01, ###*p* < 0.001. Data represented as mean ± standard deviation].

To investigate the influences of CLCA2 on cardiomyocyte cell cycle activity, CLCA2 knock-down was performed by CLCA2 siRNA. No observable differences were found in cell morphology and beating after 48 h CLCA2 siRNA treatment. The effectiveness of CLCA2 siRNA was examined by western blot (Figure 7A). A 60% decrease in mature CLCA2 protein (141 kDa) level was observed 2 days after transfection (Figure 7B). Cardiomyocyte cell cycle activity was then examined by BrdU incorporation in CLCA2 knock-down cardiomyocytes (Figure 7C). Lowering CLCA2 level increased the percentage of BrdU positive cardiomyocytes relative to control in a manner comparable to fetal dECM treated primary cells. The results indicate that CLCA2 suppression can activate cardiomyocyte cell cycle activity.

## Discussion

In this study, we demonstrate that modulating heart microenvironment stiffness alters dECM-induced cardiac regeneration in non-regenerative juvenile mice challenged with a myocardial infarction. We identify CLCA2 as a potential signaling pathway mediating dECM induced cardiomyocyte cell cycle activity. Previous studies have demonstrated that dECM derived from neonatal mouse hearts prevents adult mouse cardiac remodeling after MI (27), that lowering heart stiffness reduces post-ischemic fibrosis in mice (8), and that dECM derived from regenerative hearts promotes cardiomyocyte proliferation (21, 26). Nevertheless, we demonstrate in this study that cellular responses to extracellular factors that stimulate heart regeneration and reverse MI signaling in low-regenerative mammalian hearts function in a mechanosensitive manner. The results are clinically relevant by indicating that mechanical properties of the heart influence the therapeutic efficacy of extracellular matrix proteins-derived drugs for heart attack, and revealing a potential target for heart injury treatment.

Mechanical properties of the microenvironment have been shown to regulate cell physiology and morphology. Stiffening microenvironment stimulates fibroblast to myofibroblast activation (45–47). Modulating microenvironment stiffness has been shown to influence cardiomyocyte proliferation and maturation, and stem cell lineage (13, 15, 48–50). The role of microenvironment stiffness on heart regeneration has not been fully understood. The elastic modulus of the rodent heart increases by 3-folds with natural aging to adulthood and increases further with disease (9, 12, 51). Lowering stiffness does not increase regeneration directly in our hands; though we show altering stiffness had anticipated effects on fibrosis by fibroblast activation. Our previous work has shown that cardiomyocyte cell cycle activity is sensitive to the combined treatment of tuning microenvironment stiffness and dECM hydrogel *in vitro* (16). The results of this study suggest that microenvironment stiffness alone influences fibrosis and neovascularization in post-MI juvenile mice.

BAPN has been used by other investigators to lower tissue stiffness (8, 52). At low concentrations, BAPN is reported to be safe with no direct effects on cell viability and metabolic function (53–55). In contrast, long-term administration of high doses of BAPN leads to aortic aneurysm (56, 57). There is no indication from the functional and histological analysis of this study that the transient low dose BAPN treatment altered the heart post-injury response. Genipin is a protein crosslinker that has been applied in animals with no known side effects in the heart to our knowledge. At the low dose applied in this study, we did not observe significant change in body weight after 4-day administration. In one report, genipin inhibits uncoupling protein 2 (UCP2), and UCP2 affects cardiac cells responding to oxidative stress (58). However, knock-down UCP2 did not change heart function relative to control in post-MI rats (59). Genipin has recently been reported to polarize macrophages at high concentrations leading to collagen deposition; however, we do not observe increases in interstitial fibrosis before MI or 3 days after MI (60). Also, genipin is metabolized rapidly such that genipin injected at 50 mg/kg into adult rats is almost undetectable after 60 min (61). Thus, genipin is unlikely to directly affect cardiac post-injury response 24 h after injection at 10 mg/kg. Based on the published works, we conclude that BAPN and genipin at the doses we used did not directly affect cardiac post-injury response, and the observed cardiac post-injury responses are induced by tuned microenvironment stiffness. Nevertheless, BAPN and genipin might affect other physiological processes which in turn change heart post-injury response. Rigorous characterization of blood cells and immune response to BAPN and genipin which was not a focus of this study will provide a more precise evaluation for potential direct impact on cardiac biology and heart regeneration.

Decellularized ECM has been investigated as a naturally derived biomaterial for tissue engineering and heart regeneration (62, 63). Our pilot experiment show that dECM distributes in ventricle wall within 8 h after intramyocardial injection. Injected dECM hydrogel persists *in vivo* for at least 2 weeks (64). The mechanism of dECM-induced heart regeneration remains at an early stage of investigation. What we do know is that tissue decellularization methods retain both large extracellular structural proteins and native growth factors. Our previous work indicates a higher agrin level in fetal dECM than adult dECM (16). Agrin has been reported to promote heart regeneration (30). In this study, we demonstrate that fetal porcine dECM preserves cardiac function and promotes cardiomyocyte cell cycle activity in juvenile mice. The therapeutic efficacy of fetal dECM can be further improved by lowering microenvironment stiffness. Transcriptome analysis shows that extracellular matrix organization, cell-matrix interaction, and immune response are modulated by the treatment. Identification of specific molecular factors in dECM will strengthen our finding of unique signaling pathways affected by dECM treatment. Comparative analysis of adult and fetal dECM as investigated by some groups could provide a strategy for defining putative regenerative factors. Adult cardiac dECM has been shown to benefit adult pigs after ischemic heart injury (24). In contrast to some studies, adult cardiac dECM did not improve cardiac post-injury response in an adult MI mouse model (27). We also observed that adult dECM does not stimulate cardiac repair in juvenile mice. Species differences may account for some of the differences. In addition, experimental differences such as size of infarct relative to exogenous dECM treatment volume, viscosity of the dECM, and tissue permeability may reduce retention of the dECM in mice relative to pig. The proliferation markers (PHH3, Ki67, and BrdU) employed in the study identifies cell cycle re-entry which does not equate to cardiomyocyte proliferation because of multi-nucleation and polyploidy in mature cardiomyocytes. Accurate quantification of proliferative cardiomyocytes requires more rigorous methods such as serial histology for individual cell mapping, isotope labeling for imaging mass spectrometry, and time-lapse microscopy in culture. Immune response is an important factor that regulates cardiac post-injury response though not the focus of this study. It has been reported that monocytes density was reduced by (murine) early-aged cardiac dECM treatment in adult mice hearts 6 weeks post-MI compared to MI control and adult dECM treatment (27). Further analysis will determine the impact of mechano-tuned dECM on the cardiac post-injury immune response.

Chloride channel accessory 2 (CLCA2), is a member of family of calcium-dependent chloride channel regulators with diverse cellular actions including cell adhesion, migration, cell cycle, apoptosis, and blood pressure (40–42, 65, 66). It has been shown that CLCA2 expression can be increased by p38/c-Jun N-terminal kinase (JNK) and p53 in response to cell stress (40, 67). Lowering CLCA2 level increases the proliferation of cell lines (41, 68). While CLCA2 has not been evaluated directly in cardiac physiology, a missense mutation in CLCA2 is associated with cardiac conduction block (69). The function of CLCA2 in heart post-MI injury response is not clear. Our data suggest that CLCA2 expression is stimulated by myocardial infarction and is sensitive both to fetal dECM factors and tissue stiffness. Given the participation of stretch activated ion channels in cardiac signaling such as Piezo1 and TRPC1 (33), CLCA2 is an intriguing possibility as a regulatory protein of mechano-signaling. Myocardial infarction did not significantly change CLCA2 expression though it was interest for the inverse response to the dECM treatments and potential mechano-signaling. Using primary cardiomyocytes cultured in dish, we observed that knockdown of CLCA2 expression promoted cardiomyocyte cell cycle activity. The function of CLCA2 in cardiomyocytes has not been investigated. Studies of Ca^2+^-sensitive Cl^−^ channel (CLCA) proteins reveal that CLCA proteins play a functional role in pulmonary vein, norepinephrine-induced cardiac automaticity and membrane ion flow (34–36). How microenvironment stiffness affects CLCA2 expression and how CLCA2 modulates cardiomyocyte cell cycle activity are to be determined with future *in vitro* and *in vivo* experiments. We observed only slight trends for tissue stiffness effects on CLCA2 expression. More direct experiments will interrogate the role of mechano-stimuli on CLCA2 activity and its role in cardiomyocytes and endothelial cells.

In our previous studies, we investigated how decellularized matrix treatments and tuning tissue stiffness affect cardiomyocyte cell cycle activity, fibroblast activation, and angiogenesis in an *in vitro* explant model derived from regenerative mice (16, 28). This study extends the previous *in vitro* works to an *in vivo* model of MI in non-regenerative mice. This work also provides a more comprehensive view of dECM and microenvironment stiffness induced heart repair by examining cardiac function and cell phenotypes at different time points and with more experimental treatments. The clinical relevance is reinforced by employing juvenile mice MI model which exhibit permanent scarring and functional loss similar to adult MI models, and recapitulate aspects of pediatric derived tissue that show sensitivity to regenerative molecular programming (70). In addition, we used a transcriptome dataset previously screened for fibrotic signaling. Here, we continue this strategy to screen for novel cardiomyocyte-specific signaling. To confirm the differential expression of proteins identified by transcriptome analysis, we evaluated post-MI hearts for protein localization by immunostaining and protein level by western blot. We identified a protein that correlates with fetal dECM-induced cardiomyocyte cell cycle activity. Future studies will interrogate the combined effects of dECM and mechano-stimuli on cardiac post-injury response in aged animal MI model, CLAC2 mechanosensitivity and CLCA2 function in cardiomyocyte proliferation.

This study is not intended to suggest a new prophylactic therapy preceding a heart attack such as ibuprofen, instead, it is to investigate the interaction of mechanical and biomolecular cues in cardiac repair. This study indicates that microenvironment stiffness at the time point of myocardial infarction influences the effectiveness of a matrix proteins-derived therapy. Future studies employing treatment in large animal MI models and evaluating CLCA2 expression in biopsies of mechanically unloaded hearts (e.g., before and after ventricular assist devices) will demonstrate clinical relevance. Understanding the factors that regulate heart regeneration will benefit the development of therapeutic strategies for heart disease patients. Because fibrosis and ventricle remodeling can increase infarct area tissue stiffness following ischemic heart injury, the effectiveness of heart disease therapies can be dampened and will require consideration in the development of regenerative therapies for the aging population.

## Data Availability Statement

The datasets generated during the current study are available from the corresponding author by reasonable request. mRNA sequencing data can be accessed at https://www.ncbi.nlm.nih.gov/sra/PRJNA768462.

## Ethics Statement

The animal study was reviewed and approved by Institutional Animal Care and Use Committee, Case Western Reserve University.

## Author Contributions

XW and SES designed the research. XW and VP performed the surgeries and echocardiography. XW, VP, and SS performed the research and analyzed the data. XW, SES, and PP wrote the paper. All authors contributed to the article and approved the submitted version.

## Funding

This work was supported by Faculty Investment Fund RES221997 from Case Western Reserve University (CWRU) (SES), NIH 1 C06 RR12463-01, and R01EY021731 (PP). We acknowledge the assistance of the CWRU SOM Light Microscopy Core Facility, NIH Grant S10-OD024996, and NIH Grant S10-OD016164 for confocal microscopy.

## Conflict of Interest

The authors declare that the research was conducted in the absence of any commercial or financial relationships that could be construed as a potential conflict of interest.

## Publisher's Note

All claims expressed in this article are solely those of the authors and do not necessarily represent those of their affiliated organizations, or those of the publisher, the editors and the reviewers. Any product that may be evaluated in this article, or claim that may be made by its manufacturer, is not guaranteed or endorsed by the publisher.
