# Supplementary material for: Microenvironment Stiffness Amplifies Post-ischemia Heart Regeneration in Response to Exogenous Extracellular Matrix Proteins in Neonatal Mice
Source: Front Cardiovasc Med. 2021 Nov 5;8:773978. doi: 10.3389/fcvm.2021.773978 (PMC8602555; doi:10.3389/fcvm.2021.773978)
Supplement: Supplementary file 1 [file Data_Sheet_1.docx]

**Supplementary methods**

**Ventricle explant culture and mRNA sequencing**

Collagen type I (Corning, Corning, NY, US) hydrogel (1.5mg/ml) was prepared following manufacturer’s protocol. After polymerization in 48-well tissue culture plates, collagen gels were equilibrated in M199 (ThermoFisher) media overnight. M199 was removed before plating explants.

Newborn (day1) mouse hearts were harvested and washed in cold PBS. Ventricles were cut to approximately 1mm^3^ pieces using a surgical scissor. Two microliters of 10mg/ml fetal dECM were injected to the core of explants using a Hamilton syringe equipped with 23G needle. Explants and dECM were placed on collagen gel and incubated at 37˚C for 3h. Explants were cultured in M199 supplemented with 1% heat-inactivated fetal bovine serum (ThermoFisher), insulin-transferrin-selenium (ThermoFisher, ITS-G 100X), 2mM L-glutamine, and 100U/ml P/S (explant culture media) for 6 days. Media changed every 3 days.

Pharmacological agents altered mechanical properties of the ECM of ventricle explants. BAPN was added to explant culture media at a final concentration of 0.2mM to decrease the explant ECM stiffness. Ribose treatment at a final concentration of 5mM increased the explant stiffness. The elastic modulus of decellularized explants was measured by AFM indentation measurements.

After 6-day culture, explants were washed in 1x PBS twice and homogenized in RNA extraction buffer by bead blender. RNeasy Mini kits (QIAGEN, US) were used for RNA extraction according to the manufacturer's protocol. RNA degradation was examined by agarose gel electrophoresis, purity examined by Nanodrop (Thermo Fisher), quantification examined by Qubit (Thermo Fisher), and integrity examined by Agilent 2100 (Agilent, Santa Clara, CA, US). The mRNA was enriched by oligo beads, fragmented randomly, and used for cDNA synthesis. After purification and enrichment, cDNA was sequenced using HiSeq systems (Illumina, San Diego, CA, US). Reference genome and gene model annotation files were downloaded from the genome website browser (NCBI/UCSC/Ensembl) directly. Indexes of the reference genome were built using STAR and paired-end clean reads were aligned to the reference genome using STAR (v2.5). HTSeq v0.6.1 was used to count the read numbers mapped of each gene. And then FPKM of each gene was calculated based on the length of the gene and reads count mapped to this gene. Differential expression analysis between two conditions/groups was performed using the DESeq2 R package (2_1.6.3). The resulting P-values were adjusted using the Benjamini and Hochberg’s approach for controlling the False Discovery Rate (FDR). Genes with an adjusted P-value < 0.05 were assigned as differentially expressed. Differentially expressed genes between the control and fetal dECM treated samples were categorized by Gene Ontology (GENEONTOLOGY, the Gene Ontology) [71], [72]. Row z-scores were calculated in MATLAB (MathWorks) and a heatmap was generated using MATLAB.

**Supplementary Figures**

**
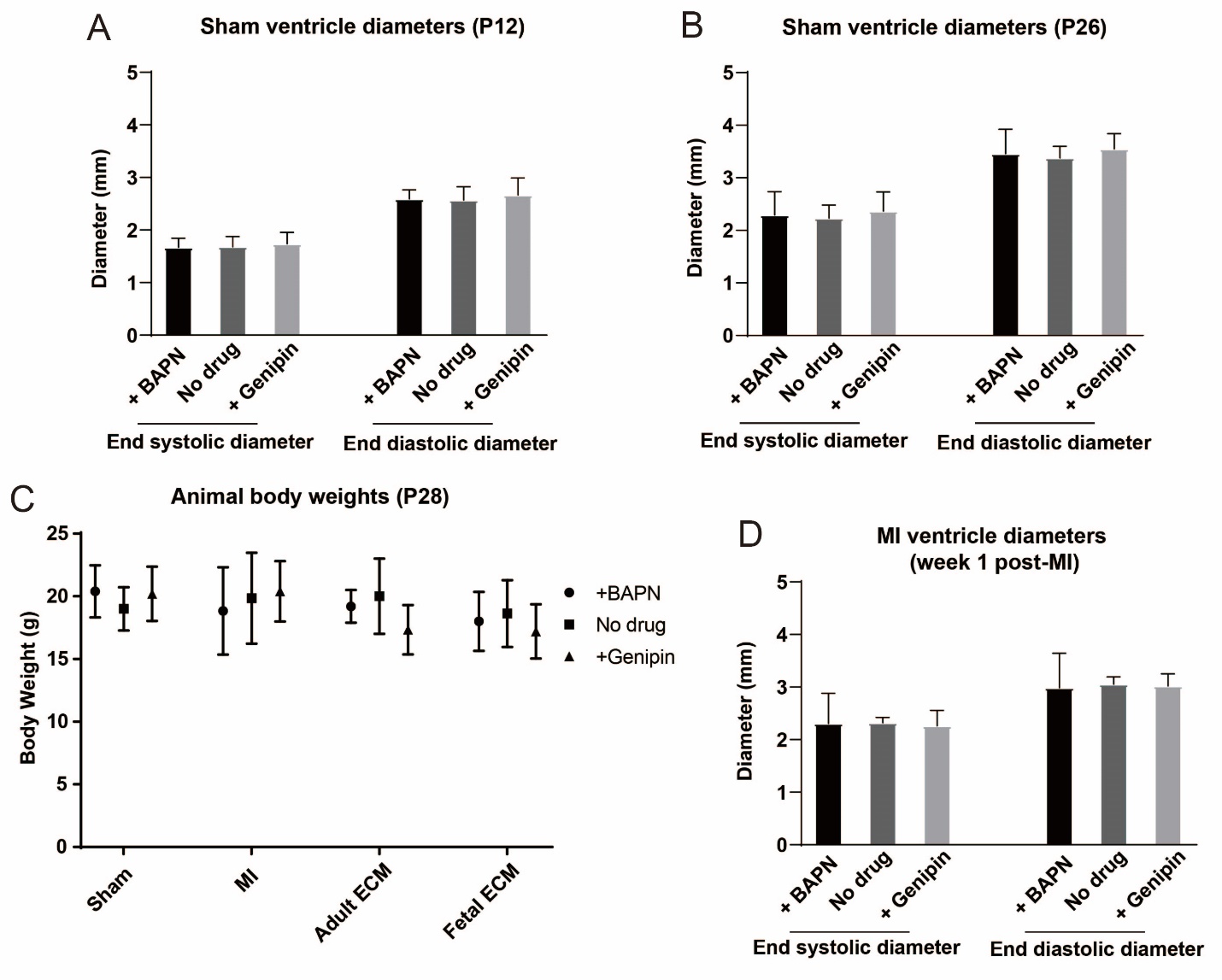
**

**Supplement figure 1. BAPN and genipin did not change left ventricle diameters and body weights in mice.** (A) Sham hearts left ventricle systolic and diastolic diameters measured by echocardiography at week 1 post-surgery. (B) Sham hearts left ventricle diameters at week 3 post-surgery. (C) Animal body weights at week 3 post-surgery. (D) MI heart left ventricle systolic and diastolic diameters measured by echocardiography at week 1 post-surgery. (n=5, one-way ANOVA and Tukey’s test. Data represented as mean ± standard deviation.)


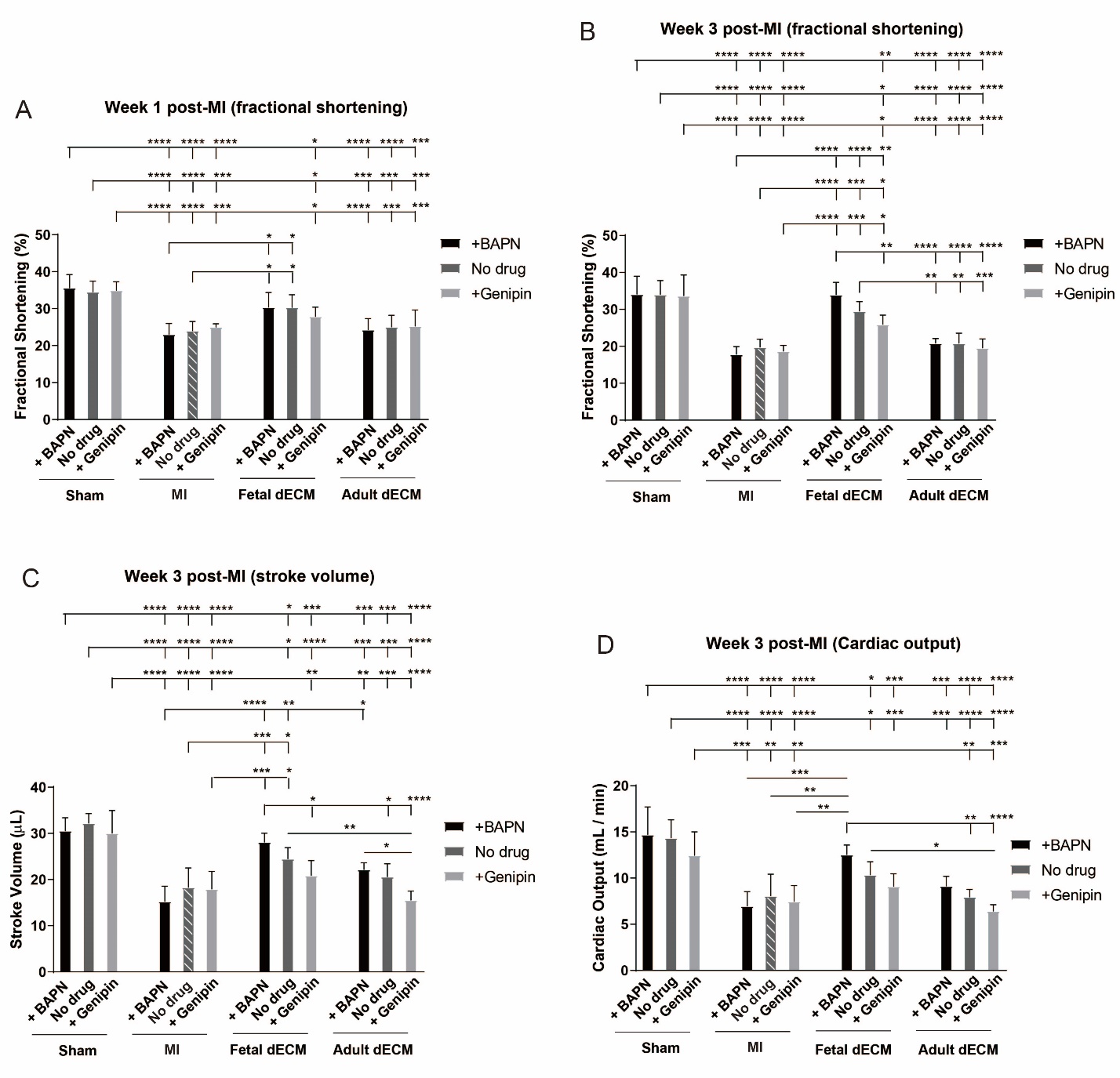


**Supplement figure 2. Cardiac function at week 1 and week 3 post-MI.** (A) Fractional shortening at week 1 post-surgery. Fetal dECM increased fractional shortening in BAPN-treated and untreated MI hearts but not in genipin-treated hearts compared to MI-no drug at week 1. (B) Fractional shortening of sham and MI groups at week 3 post-surgery. Fetal dECM treated hearts showed increased fractional shortening at week 3 compared to the MI-no drug which increased in combination with BAPN softening. (C) Stroke volume at week 3 post-surgery. Fetal dECM therapeutic efficacy can be improved by lowering heart stiffness. (D) Cardiac output at week 3 post-surgery. (Panel A, B, C, D: n=5, two-way ANOVA and Tukey’s test, *p<0.05, **p<0.01, ***p<0.001, ****p<0.0001. Data represented as mean ± standard deviation. The lines above the plot represent the statistical significance between the tick-designated first group with the following ticks. The line without ticks represents the statistical significance of only two groups.)


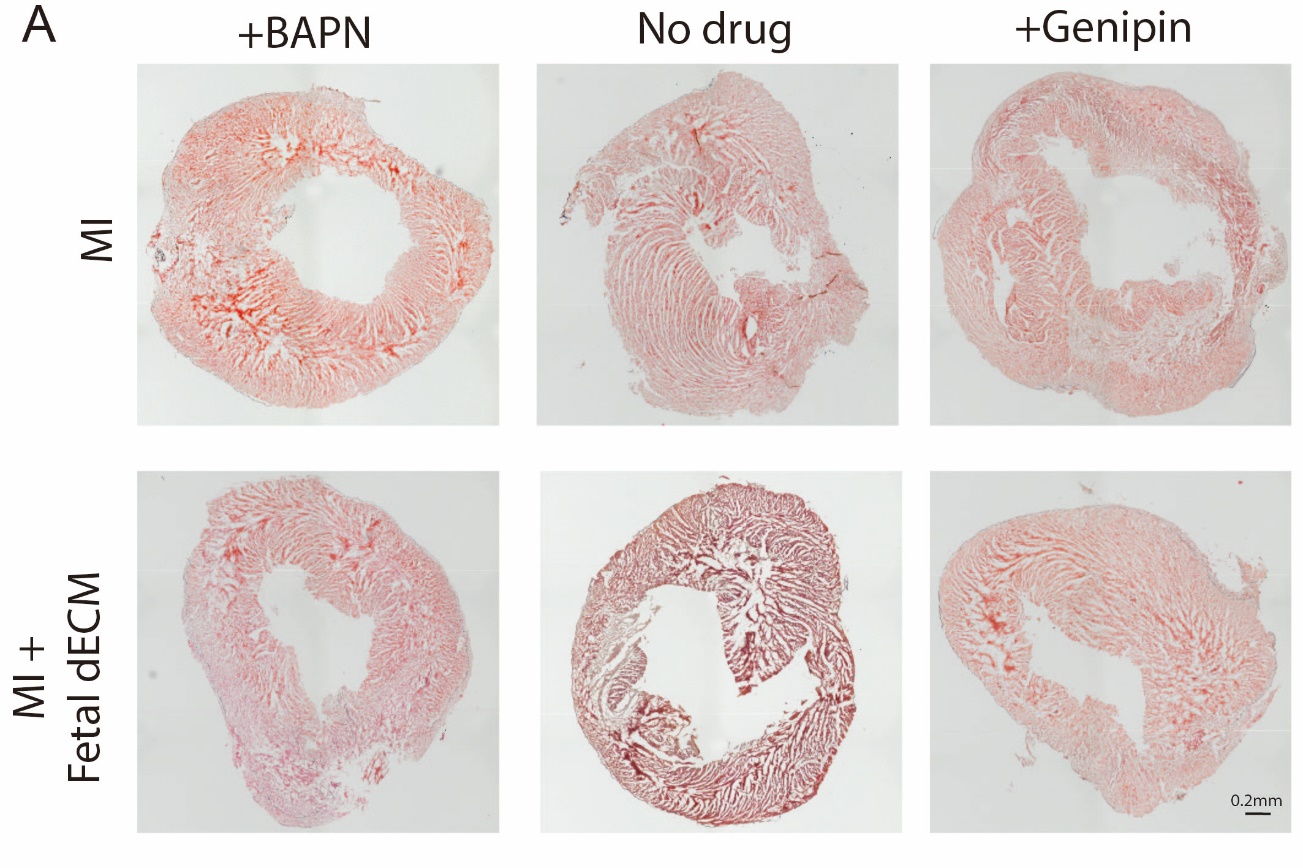


**Supplement figure 3. Masson’s Trichrome staining of hearts on day 3 post-surgery.** No fibrotic tissue was observed in 3 days post-surgery hearts. MI surgery and protein crosslinking modulation did not result in observable morphological change on day 3.


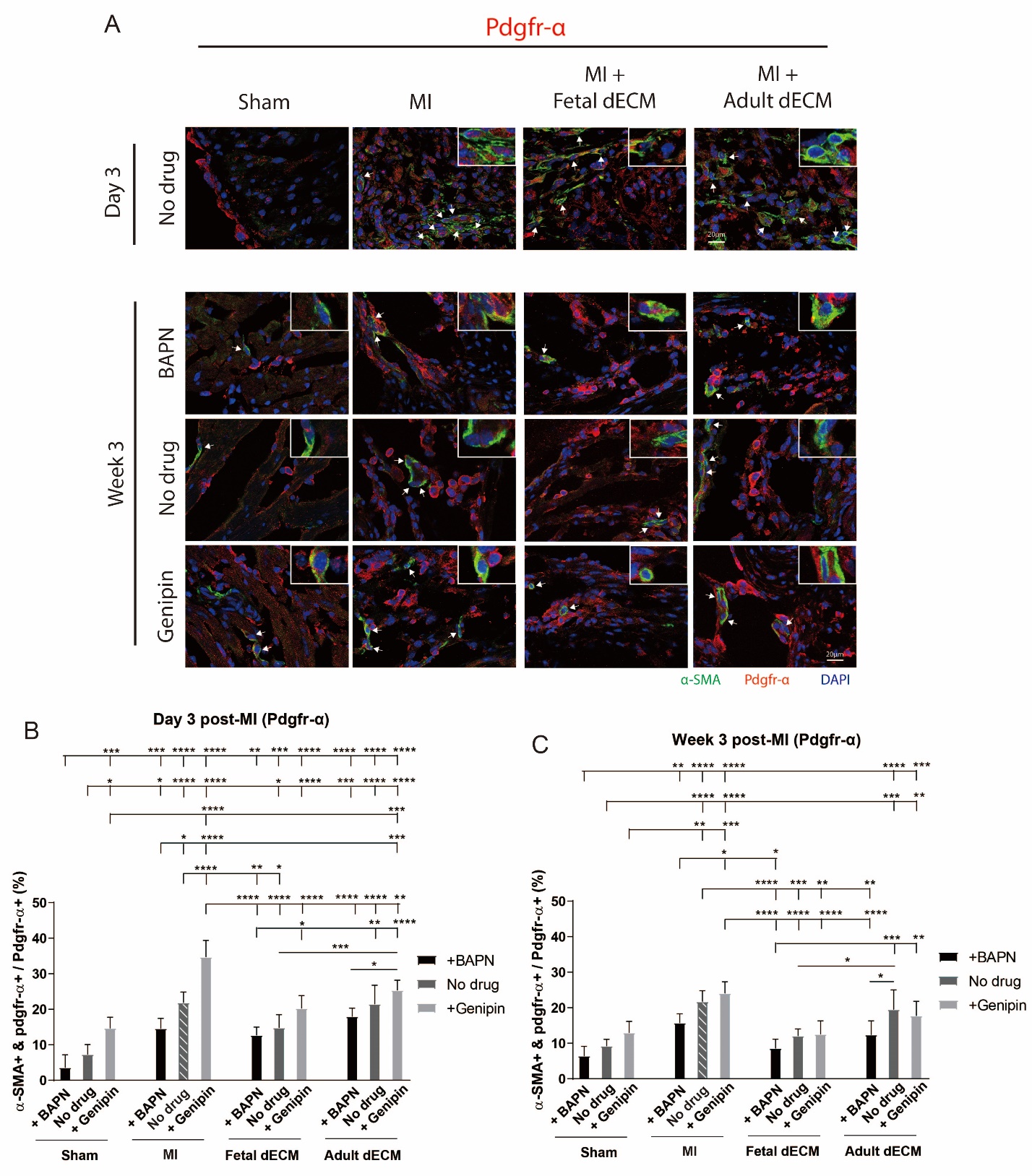


**Supplement figure 4. Decreasing microenvironment stiffness reduced α-SMA expression in Pdgfr-α positive cells.** (A) Heart sections were immunostained for α-SMA and Pdgfr-α to examine fibroblast activation. (B) Day 3 quantification of α-SMA and Pdgfr-α double positive cells. Stiffening hearts by genipin stimulates fibroblast activation. Fetal dECM lowered fibroblast activation in MI hearts on day 3 post-MI with further decreases with softening. Adult dECM did not significantly change fibroblast activation compared to MI-no drug. (C) Week 3 quantification of double positive cells. Fetal dECM lowered fibroblast activation compared to the MI-no drug on week 3 at all stiffness levels. BAPN-softening treatment lowered activated fibroblasts in adult dECM treated hearts relative to MI-no drug. (Panel B, C: n=5, two-way ANOVA and Tukey’s test, *p<0.05, **p<0.01, ***p<0.001, ****p<0.0001. Data represented as mean ± standard deviation. The lines above the plot represent the statistical significance of the tick-designated initial group with the following ticks. The line without ticks represents the statistical significance of only two groups.)


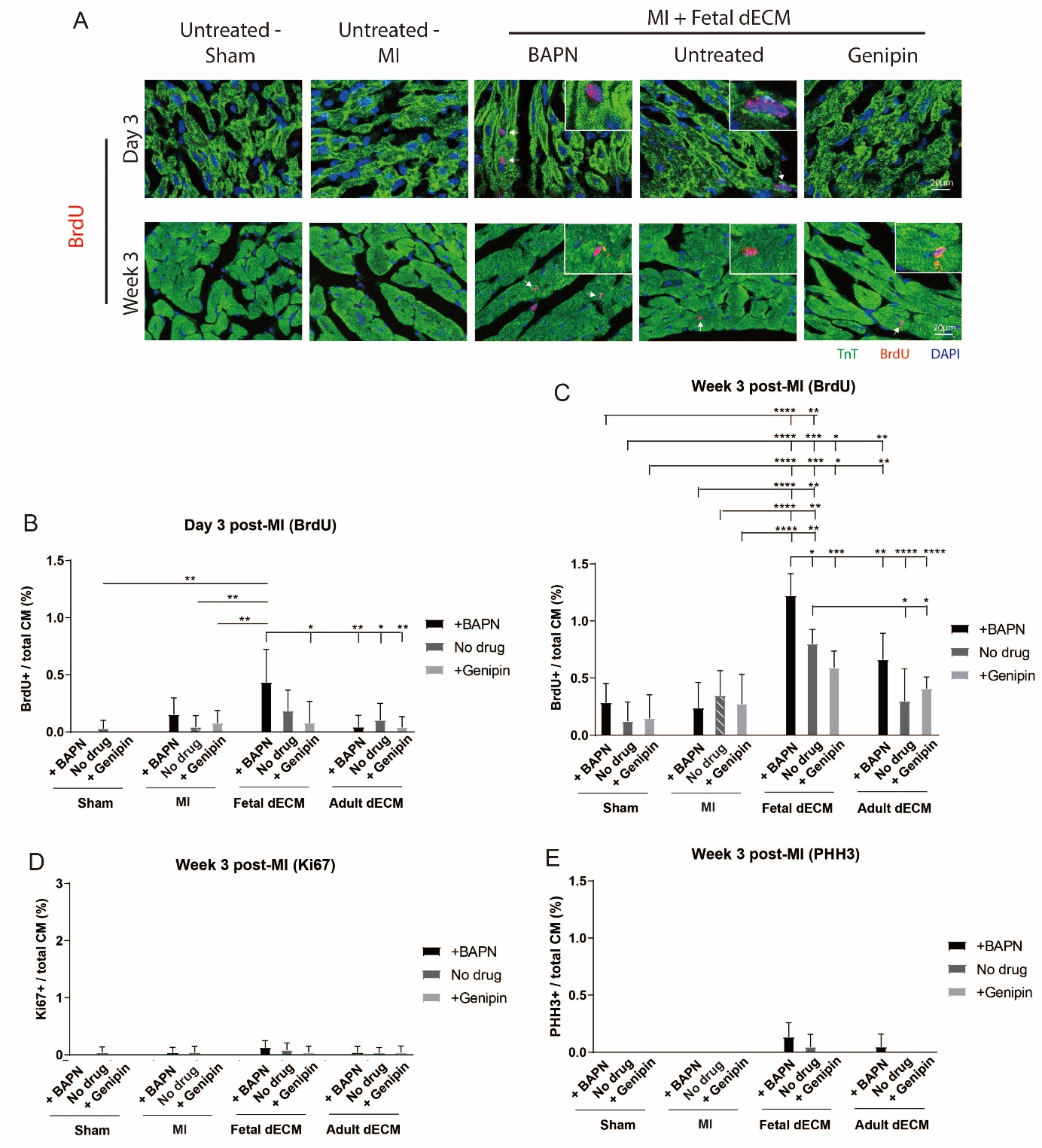


**Supplement figure 5. BrdU incorporation and cell cycle activity markers expression in cardiomyocytes.** (A) BrdU incorporation in cardiomyocytes was examined by immunostaining. (B) BrdU incorporation in cardiomyocytes on day 3 post-surgery. Cell cycle activity was labeled by BrdU for 12h. Softening hearts by BAPN increased BrdU incorporation in fetal dECM treated animals. (C) BrdU incorporation in cardiomyocytes at week 3 post-surgery. Cell cycle activity was labeled by BrdU for 2 days. Fetal dECM increased cardiomyocyte BrdU incorporation compared to MI control. Lowering heart stiffness promoted BrdU positive cells population in fetal dECM treated hearts. (D) Ki67 positive and (E) PHH3 positive cardiomyocytes quantifications at week 3 post-surgery. No difference was observed across groups. (Panel B, C, D, E: n=5, two-way ANOVA and Tukey’s test, *p<0.05, **p<0.01, ***p<0.001, ****p<0.0001. Data represented as mean ± standard deviation. The lines above the plot represent the statistical significance of the tick-designated initial group with the following ticks. The line without ticks represents the statistical significance of only two groups.)


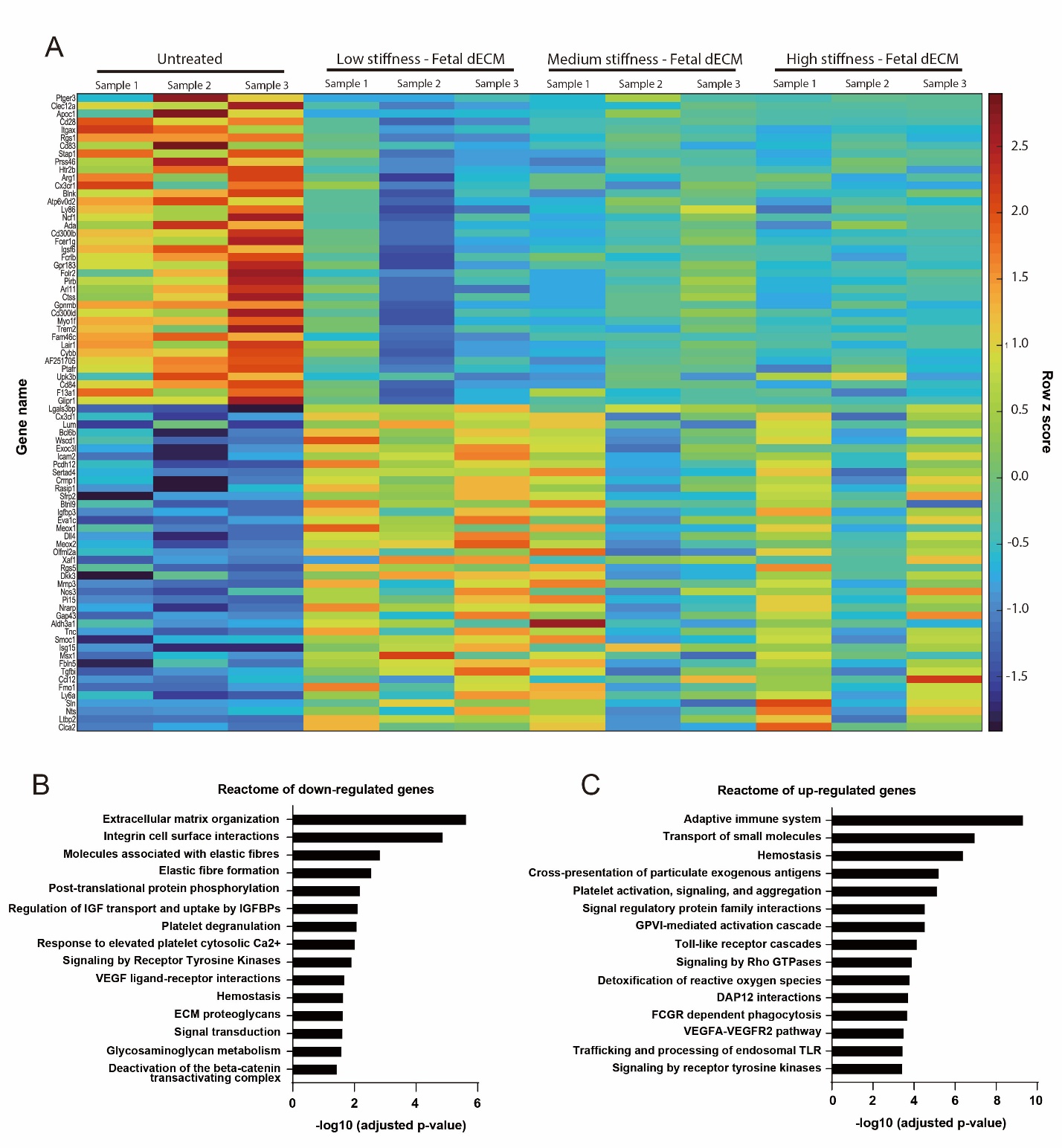


**Supplement figure 6. Modulating microenvironment stiffness changed mRNA transcription in fetal dECM treated heart explants.** The transcriptome of fetal dECM treated cardiac explants of different stiffness was analyzed. (A) heatmap of top 40 up- and down-regulated genes in fetal dECM treated heart explants of different stiffness versus normal-stiffness no-dECM treatment control. (B) Reactome of down-regulated genes. (C) Reactome of up-regulated genes.


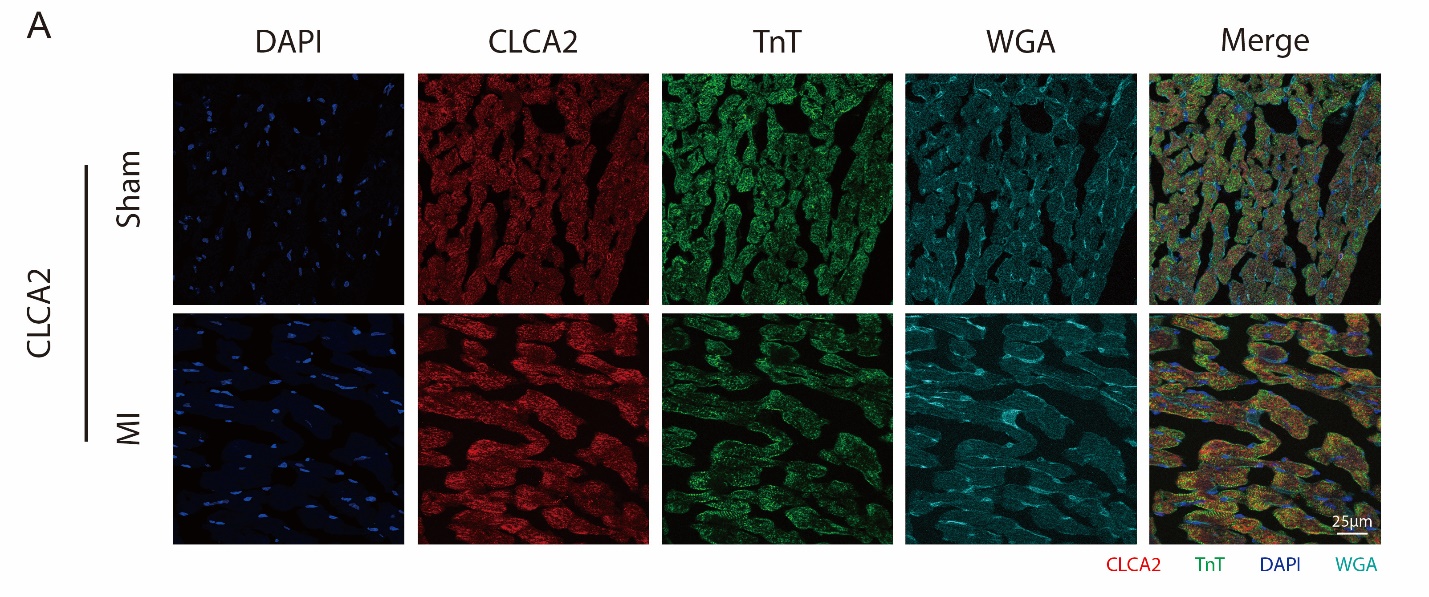


**Supplement figure 7. CLCA2 mainly distribute in cardiomyocytes.** (A) CLCA2 distribution in cardiac cells was examined by immunostaining. CLCA2 was distributed primarily in cardiomyocyte membrane and cytoplasm.
